# Analyzing Fluoride Binding by Group 15 Lewis Acids: Pnictogen Bonding in the Pentavalent State

**DOI:** 10.1021/acs.inorgchem.3c01987

**Published:** 2023-08-08

**Authors:** Logan T. Maltz, François P. Gabbaï

**Affiliations:** Department of Chemistry, Texas A&M University, College Station, Texas 77843, United States

## Abstract

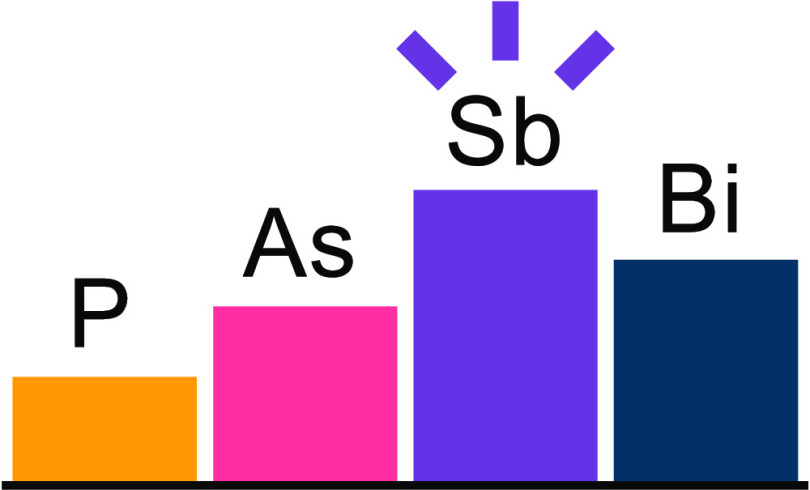

We report the results of a computational investigation into fluoride binding by a series of pentavalent pnictogen Lewis acids: pnictogen pentahalides (PnX_5_), tetraphenyl pnictogeniums (PnPh_4_^+^), and triphenyl pnictogen tetrachlorocatecholates (PnPh_3_Cat). Activation strain and energy decomposition analyses of the Lewis adducts not only clearly delineate the electrostatic and orbital contributions to these acid–base interactions but also highlight the importance of Pauli repulsion and molecular flexibility in determining relative Lewis acidity among the pnictogens.

## Abstract 2

Among pentavalent pnictogen Lewis acids, antimony-based species stand out as the strongest fluoride binders. Computational activation strain and energy decomposition analyses highlight the importance of Pauli repulsion and molecular flexibility in determining this relative Lewis acidity.

## Introduction

Among Lewis acids, antimony holds a special place. SbF_5_, in particular, is a Lewis superacid^1^ that has had profound impacts on chemistry as exemplified by the work of Olah involving magic acid.^2^ Recently, our group^3^ and others^4^ have effectively employed the unique Lewis acidity of Sb to develop transmembrane anion transporters and anion-recognition platforms. But what is it that distinguishes Sb from the other elements in the pnictogen (Pn) group? As chemists, we turn to chemical bonding and the competition between covalency and ionicity to answer this question.

Being saturated or hypervalent, pentavalent pnictogens use an empty σ*-orbital to accept electron density. At the same time, the coincident σ-hole provides Coulombic stabilization to the newly formed linkage. Scheiner details the importance of these effects in his original definition of the pnictogen bond using trivalent elements^5^ which has since expanded to include the interactions between any pnictogen-based Lewis acid—in the trivalent or pentavalent state—and a Lewis base.^6^ Obviously, the distinction between the pnictogens must rely on amplification of whichever form of bonding predominates. Is the interaction more covalent? Then we might look to relative lowest-unoccupied molecular orbital (LUMO) energies to provide insight into the increasing Lewis acidity down the group.^7^ Does ionicity dominate the bonding interaction? Then we might look to measures of the electrostatic potential to understand the increased Lewis acidity of Sb derivatives.

Wanting simple, intuitive descriptions of chemical bonding, we sometimes forget its complexity. Fortunately, chemists have developed models to better conceptualize complex interactions. Computational energy decomposition analysis (EDA) provides a convenient way to break an interaction into various energetic contributions: London dispersion interactions (Δ*E*_disp_), electrostatic interactions (Δ*E*_el_), orbital interactions (Δ*E*_oi_), and Pauli repulsion (Δ*E*_Pauli_). In our constant debates about the covalency or ionicity of an interaction, we often neglect London dispersion and Pauli repulsion.

Hypervalent SbF_5_ reminds us that with any interaction—but especially closed-shell interactions—we need to consider Pauli repulsion: the destabilizing interaction occurring when two filled orbitals interact with each other. This repulsion is the underlying electronic basis for what we term “steric interactions” and is also at play in our discussions of ionic and covalent bonding. In this paper, we contend that Pauli repulsion rivals electrostatic and orbital interaction contributions in its importance to the Lewis acidity of the pnictogens.

**Figure 1:**
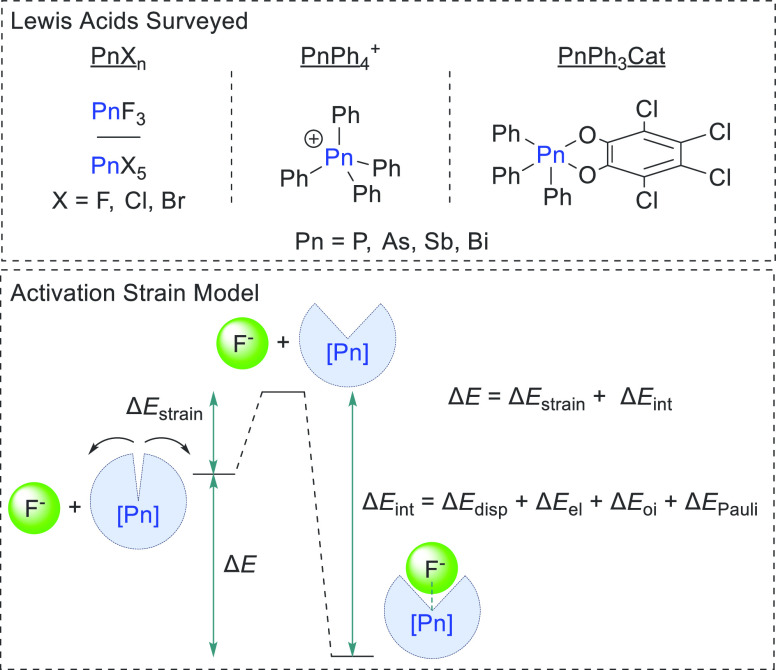
Top: Lewis acids surveyed in this study. Bottom: diagram of the activation strain model and the energy components comprising the overall interaction energy between the Lewis acids studied and F^–^.

In the past decade, the utility of the activation strain model (ASM) has been repeatedly demonstrated.^8^ This model bifurcates the overall interaction energy Δ*E* into the energy necessary to strain and reorganize the interacting species into their interacting geometries (Δ*E*_strain_) and the energy associated with allowing these strained species to interact (Δ*E*_int_).^8a^ To fully understand the interactions in these systems, Δ*E*_int_ is then parsed into its constituent components using EDA in the Amsterdam Density Functional (ADF) program (Figure 1). This method conveniently captures Δ*E*_strain_ and Δ*E*_Pauli_ which are important components of the overall interaction energy that are often overlooked because they are not as comfortably approachable as Δ*E*_el_ and Δ*E*_oi_.

Inspired by Bickelhaupt and co-workers’ analysis of trivalent pnictogen trihalides,^9^ we have undertaken a similar analysis on a series of pentavalent pnictogen Lewis acids: pnictogen pentahalides (PnX_5_), tetraphenyl pnictogeniums (PnPh_4_^+^), and triphenyl pnictogen tetrachlorocatecholates (PnPh_3_Cat) (Figure 1). The last two families of compounds were selected because of their extensive use by our group as anion-binding platforms, anion sensors, and anion transporters.^3^ Unlike the previous work on trivalent pinctogens,^9^ we expanded the scope of Lewis acids beyond the homoleptic halides but narrowed the scope of Lewis bases, focusing on these acids’ interactions with fluoride (F^–^). As such, we are effectively decomposing fluoride ion affinities (FIAs), though we are assessing changes in electronic energy (Δ*E*) while FIAs are defined as changes in enthalpy (Δ*H*).

The computations and analyses presented in this article illustrate that despite having lower magnitudes of stabilizing contributions from Δ*E*_el_ and Δ*E*_oi_, Sb displays the highest Lewis acidity (most negative Δ*E*) in almost every case analyzed, the only exception being the trivalent pnictogen trifluorides. This result is due to Sb also having lower magnitudes of destabilizing contributions from Δ*E*_strain_ and Δ*E*_Pauli_.

## Computational Methods

For computational efficiency, we optimized the initial geometries of the Lewis acids and their fluoride adducts in Orca 5.0.2^10^ using PBEh-3c/def2-mSVP^11^ with the default defgrid2 settings. Frequency calculations were performed at the same level of theory to verify that all optimized structures were at a local minimum on the potential energy surface. Natural population analysis (NPA) charges were obtained through Natural Bonding Orbital calculations using NBO 7.0 at the same level of theory.^12^ Where possible, structures were reoptimized from previously optimized coordinates.^9,13^ All other structures were initially produced using either GaussView 6.1.1^14^ or Avogadro^15^ or by substituting one atom for another in the input file before performing the optimization depending on which method was simpler. For the F^–^ adducts of the PnPh_3_Cat species, two isomers were possible: F *trans* to Ph or F *trans* to O in the tetrachlorocatecholate. In the main text, the isomer with F *trans* to Ph is discussed as it is the lowest-energy isomer for Sb and similar trends are seen among both isomers. For completeness, both isomers were fully analyzed, and that data is presented in Table S1 and Graphs S7–S9.

The structures optimized in Orca were used as inputs for single-point energy calculations and EDA^16^ computations conducted in ADF 2022.101^17^ using the M06 functional^18^ paired with the D3 model to account for dispersion effects.^19^ The QZ4P basis set^20^ as implemented in the ADF program was used without frozen-core approximation and with good numerical quality. The zeroth-order regular approximation (ZORA) Hamiltonian was employed to account for scalar relativistic effects.^21^ To avoid numerical issues, the “Fix Dependencies” function in ADF was enabled for the PnPh_4_^+^ and PnPh_3_Cat species due to their size. Δ*E*_strain_ was determined by subtracting the single-point energy of the free Lewis acid from the single-point energy of the strained Lewis acid with no F^–^ bound. EDA directly provided Δ*E*_disp_, Δ*E*_el_, Δ*E*_oi_, and Δ*E*_Pauli_. LUMO energies were obtained from ADF as well.

Because EDA divides the Lewis adduct into its constituent acid and the small, highly negative F^–^ base, we used the counterpoise method as implemented in ADF to investigate the basis set superposition error (BSSE).^22^ The BSSE was determined to be in the narrow range of 2.88–3.74 kcal mol^–1^ for all species, predominantly due to F^–^, with the Lewis acid contributing ≤0.3 kcal mol^–1^ to the BSSE in all cases. In accordance with prior EDA investigations of main group Lewis acid adducts,^9,23^ the individual BSSEs were not incorporated in the reported energy values. As expected for a hard ion such as F^–^, Δ*E*_disp_ is negligible for all Lewis acids considered, reaching a maximum magnitude of −0.5 kcal mol^–1^ in the PnPh_4_^+^ and PnPh_3_Cat species which is expected given their larger surface areas (Table S1).

## Results and Discussion

**Chart 1:**
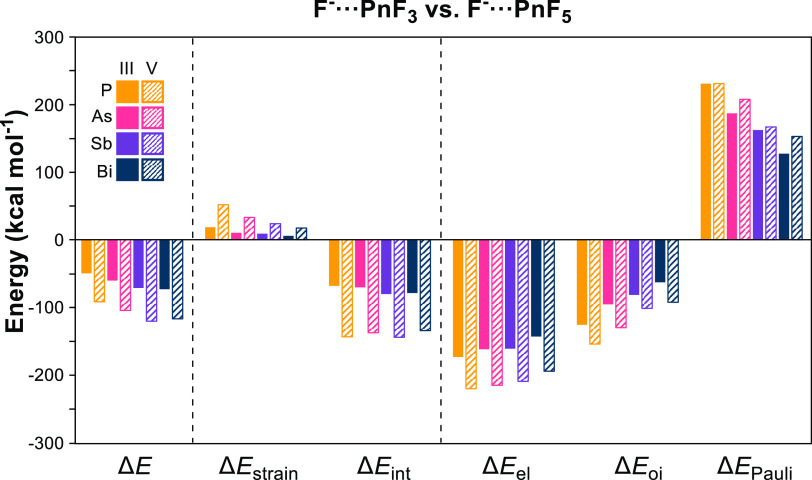
Bar Graph Depicting the Data from the Activation Strain and Energy Decomposition Analyses of the F^-^···PnF_3_ and F^-^···PnF_5_ Seriesa

Our lab has previously demonstrated that oxidizing the pnictogen center from the +3 state to the +5 state increases its Lewis acidity.^3f^ This conclusion is corroborated by the ∼40 kcal mol^–1^ increase in the magnitude of Δ*E* for all pnictogens going from PnF_3_ to PnF_5_ (Table 1). Gratifyingly, this data vindicates our assertion that oxidation leads to both an increase in the electrostatic contribution to the interaction through a deepening of the σ-hole and an increase in the orbital contribution through the lowering of the σ*-based LUMO (Chart 1). Moving from PnF_3_ to PnF_5_, we also see an increase in Δ*E*_strain_ and Δ*E*_Pauli_ as expected with an increased number of substituents attached to the central pnictogen and a decrease in the bond lengths upon oxidation. Thus, for oxidation from Pn^III^ to Pn^V^, the substantial increase in stabilization energy leads to greatly enhanced Lewis acidity despite a simultaneous increase in destabilizing interactions. As we will discuss, this scenario is inverted when looking at the periodic trends across the pentavalent pnictogens.

a Δ*E*_disp_ has been omitted for clarity.

**Table 1:** Activation Strain and Energy Decomposition Analyses (in kcal mol^–1^) at Optimized Geometries^a^

Acid	Δ*E*	Δ*E*_strain_	Δ*E*_int_	Δ*E*_el_	Δ*E*_oi_	Δ*E*_Pauli_	*d*_Pn···F_ (Å)	Charge^b^	*E*_LUMO_ (eV)^c^
F^–^···PnF_3_									
PF_3_	–49.6	18.5	–68.1	–173.2	–125.6	230.7	1.738^d^	1.77	–2.16
AsF_3_	–60.1	10.2	–70.4	–161.9	–95.3	186.8	1.847^d^	1.84	–2.50
SbF_3_	–71.2	9.0	–80.2	–161.0	–81.5	162.2	1.989^d^	1.98	–3.03
BiF_3_	–72.9	5.8	–78.7	–142.9	–62.9	127.1	2.119^d^	2.00	–2.94
F^–^···PnF_5_									
PF_5_	–91.6	51.8	–143.4	–220.1	–154.1	230.9	1.634	2.71	–5.49
AsF_5_	–104.5	33.0	–137.5	–215.1	–129.8	207.5	1.738	2.76	–6.37
SbF_5_	–120.3	23.7	–144.1	–209.3	–101.4	166.7	1.900	2.96	–6.52
BiF_5_	–116.9	17.3	–134.1	–194.2	–92.4	152.6	1.997	2.80	–7.34
F^–^···PnPh_4_^+^									
PPh_4_^+^	–125.4	33.3	–158.7	–251.1	–155.8	248.7	1.724	1.52	–5.37
AsPh_4_^+^	–123.4	23.7	–147.1	–230.8	–118.9	203.2	1.852	1.64	–5.32
SbPh_4_^+^	–142.0	18.6	–160.6	–229.4	–102.2	171.4	2.000	1.94	–5.81
BiPh_4_^+^	–138.9	14.2	–153.0	–206.1	–84.9	138.3	2.132	1.78	–6.01
F^–^···PnPh_3_Cat^e^									
PPh_3_Cat	–75.0	31.3	–106.3	–199.4	–170.2	263.8	1.683	1.76	–2.94
AsPh_3_Cat	–73.2	20.6	–93.8	–180.5	–135.3	222.5	1.801	1.89	–3.13
SbPh_3_Cat	–85.9	16.0	–101.8	–172.4	–112.9	184.0	1.955	2.23	–3.30
BiPh_3_Cat	–78.8	12.8	–91.6	–152.1	–98.7	159.6	2.065	2.05	–3.69

a Δ*E*_disp_ omitted for clarity.

b NPA charge in strained acid without F^–^.

c LUMO energy in strained acid without F^–^.

d Smaller of two Pn···F distances.

e Isomer with F *trans* to Ph. For the complete table, see Table S1 in the Supporting Information.

We focus our analysis on the PnF_5_ series as the trends seen hold for the other series. With a Δ*E* of −120.3 kcal mol^–1^—in line with previously computed fluoride ion affinities^24^—SbF_5_ is the strongest Lewis acid in this series. Down the group, there is a 28.7 kcal mol^–1^ increase in the magnitude of Δ*E* from −91.6 kcal mol^–1^ for PF_5_. This general trend of increasing Lewis acidity down the group has been observed experimentally as well.^7,25^ While the destabilization from Δ*E*_strain_ decreases from 51.8 kcal mol^–1^ for PF_5_ to 23.7 kcal mol^–1^ for SbF_5_, Δ*E*_int_ stays nearly constant, seeing only a 0.7 kcal mol^–1^ increase in magnitude.

The decrease in Δ*E*_strain_ follows from the larger size of the pnictogen center allowing increased flexibility of the coordinated ligands. This flexibility was highlighted in Moc and Morokuma’s 1997 study on hypervalent pnictogens wherein they concluded that the larger pnictogens enjoy a reduced barrier to Berry pseudorotation due to an increased ease in adjusting their Pn–F bond lengths from the ground state *D*_3*h*_ structure to achieve the transitional *C*_4*v*_ structure.^26^ Their values for the pseudorotation barrier are comparable to those calculated by Breidung and Thiel in 1992.^27^ During this conversion from *D*_3*h*_ to *C*_4*v*_, the predominantly ligand-based highest occupied molecular orbital (HOMO) decreases in energy while the pnictogen-centered HOMO–1 increases in energy.^28^ Accordingly, decreasing the destabilization of the pnictogen-based HOMO–1 corresponds with a decrease in the pseudorotation barrier. Given this analysis, it seems that the most influential factor in the PnF_5_ series is the weaker bonds formed down the group resulting from greater atomic radius and increased orbital diffuseness which both lead to less effective orbital overlap. Steric repulsion also plays a role in decreased Δ*E*_strain_ as larger atoms allow more room between the ligands as they become compressed in the *C*_4*v*_ geometry.

Turning our attention from Δ*E*_strain_, we see that though the change in Δ*E*_int_ is small down the group, the magnitude of Δ*E*_int_ is 3-6 times greater than that of Δ*E*_strain_ and thus contributes significantly to Δ*E*. As expected with increased atomic radius, Δ*E*_el_ decreases consistently down the group with SbF_5_ having an electrostatic contribution that is 10.8 kcal mol^–1^ less stabilizing than that for PF_5_. Δ*E*_oi_ sees a dramatic decrease of 52.7 kcal mol^–1^ in stabilization going from PF_5_ to SbF_5_, which can be attributed to the increased diffuseness of the pnictogen center’s orbitals leading to decreased overlap with the incoming Lewis base due to the size mismatch. This combination of increasing atomic radius and increasing orbital diffuseness progressively favors the ionic contribution down the group with Δ*E*_el_ increasing from 59% of the stabilizing contribution for PF_5_ to 67% for SbF_5_.

Despite a cumulative 63.5 kcal mol^–1^ decrease in stabilization from P to Sb, there is a simultaneous 64.2 kcal mol^–1^ decrease in Δ*E*_Pauli_ that more than compensates, producing a Δ*E*_int_ that remains largely unchanged down the group which then allows the decrease in Δ*E*_strain_ to drive the observed differences in Lewis acidity . Similar trends are seen for the pentachloride and pentabromide species as well (Supporting Information). Noticeably lacking in this discussion, however, is Bi.

While BiF_5_ is more Lewis acidic than PF_5_ and AsF_5_, there is a drop in Lewis acidity going from SbF_5_ to BiF_5_ which has also been observed experimentally and has been repeatedly reproduced in FIA calculations (Table 2).^7,25,26,29^ The trends that exist down the group still hold when going from Sb to Bi: both stabilizing and destabilizing contributions decrease. This transition, however, does not come with the same magnitude of change in the energetic contributions—the decrease in destabilizing contributions no longer compensates as much for the decrease in stabilizing contributions. While Δ*E*_el_ decreases from P to As by 2% and then from As to Sb by 3%, there is a significant 7% decrease in Δ*E*_el_ from Sb to Bi. This decrease appears less consequential upon realizing that Δ*E*_oi_ only decreases by 9% from Sb to Bi compared to a 22% decrease from As to Sb. As a result, Sb and Bi have similar ratios of Δ*E*_el_ to Δ*E*_oi_ with both having ∼32% of the stabilization energy coming from Δ*E*_oi_.

**Table 2:** Comparison of Δ*E* and FIAs (in kcal mol^–1^)

Acid	Δ*E*	FIA
PF_3_	–49.6	47.8^a^
AsF_3_	–60.1	58.3^a^
SbF_3_	–71.2	69.3^a^
BiF_3_	–72.9	
PF_5_	–91.6	91.9^b^
AsF_5_	–104.5	104.1^b^
SbF_5_	–120.3	117.6^b^
BiF_5_	–116.9	115.2^b^

a FIAs converted from kJ mol^–1^ from ref (24b).

b FIAs obtained as negatives of the reaction energy for PnF_5_ + F^–^ → PnF_6_^–^ in ref (26).

The major difference between Sb and Bi lies in the reduction of Δ*E*_Pauli_. Δ*E*_strain_ decreases rather consistently: a 28% decrease from As to Sb and a 27% decrease from Sb to Bi. This steady decrease is likely due to the predictably weaker and longer bonds formed by the more diffuse orbitals moving down the group. Δ*E*_Pauli_, on the other hand, only decreases by 8% from Sb to Bi compared to the significant 20% decrease seen from As to Sb. This inconsistency results from the unexpected trend in covalent radii. The covalent radius from As to Sb increases by 0.20 Å (1.19 vs 1.39 Å).^30^ Due to the lanthanide contraction, the increase from Sb to Bi is only 0.09 Å (1.39 vs 1.48 Å)—also reflected in the computed Pn–F bond lengths (Table 1).^30^

With a smaller-than-expected increase in size, the Bi–F bonds are closer to the incoming F^–^ than might otherwise be anticipated leading to the smaller-than-expected decrease in Pauli repulsion. As such, the larger-than-expected Pauli repulsion is not as effectively counterbalanced by the stabilizing contributions in Bi as it is in Sb, leading to a reduction in overall Lewis acidity. Owing to the scandide contraction, a similarly small decrease of 10% in Δ*E*_Pauli_ is seen for the transition from P to As; however, this 10% decrease corresponds to a considerable 23.4 kcal mol^-1^ reduction in Δ*E*_Pauli_ while the 8% drop from Sb to Bi only produces a 14.1 kcal mol^–1^ decrease, indicating that an increase in covalent radius has a more profound effect on Δ*E*_Pauli_ for smaller atoms.

**Chart 2:**
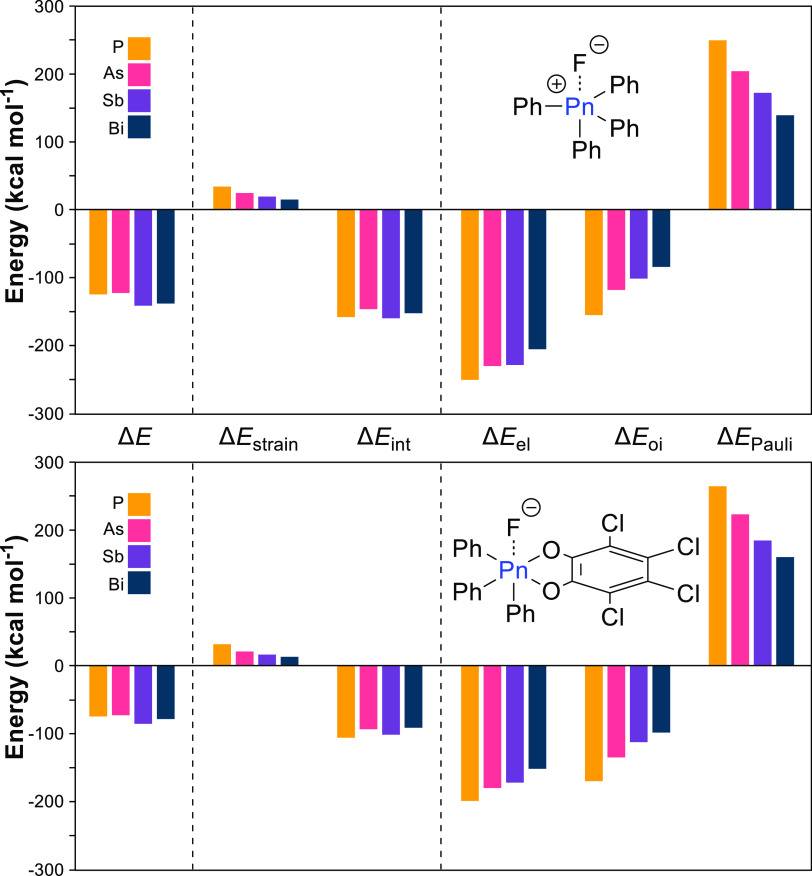
Bar Graphs Depicting the Data from the Activation Strain and Energy Decomposition Analyses of the F^-^···PnPh_4_^+^ (Top) and F^-^···PnPh_3_Cat (Bottom) Seriesa

With these trends in mind, we turn to more complex pnictogen-based Lewis acids, starting with the PnPh_4_^+^ series. These cationic species serve as representative examples of pnictogen-based Lewis acids employed extensively in anion transport.^3g^ For these cationic species—and the rest of the species studied—Δ*E* seems to oscillate: Sb and Bi have larger Δ*E*’s than P and As with Bi and As having the lower Δ*E*’s in these pairs (Chart 2). While this “secondary periodicity” is also seen in the Δ*E*_int_ of the PnF_5_ series, it likely manifests in the Δ*E* of the PnPh_4_^+^ series due to a slight increase in the importance of Δ*E*_el_ as a result of the cationic charge.^31^ The percentage of Δ*E*_el_’s contribution to the stabilization energy increases from 59–68% in the PnF_5_ series to 62–71% in the PnPh_4_^+^ series. Furthermore, Δ*E*_el_ increases in magnitude by ∼20–30 kcal mol^–1^ for P and Sb but only ∼12–16 kcal mol^–1^ for As and Bi. This observed secondary periodicity results from the scandide contraction at As and the lanthanide contraction at Bi which lead to not only smaller radii than would be expected but also higher electronegativities than expected.

a Δ*E*_disp_ has been omitted for clarity.

While electronegativity seemingly decreases down the group according to the Pauling scale, Haïssinsky reminds us that electronegativity increases with oxidation state, leading to electronegativities of 2.2 for As^V^, 2.1 for Sb^V^, and >2.3 for Bi^V^.^32^ This irregularity in the electronegativity is seen in the natural population analysis (NPA) charges in the strained geometries: +1.52 for P, +1.64 for As, +1.94 for Sb, and +1.78 for Bi (Table 1). Though there is a slight increase in charge from P to As, it cannot overcome the 0.12 Å increase in covalent radius,^30^ resulting in a large 20.3 kcal mol^–1^ decrease in Δ*E*_el_ for this pair. The transition from Sb to Bi sees an even larger decrease of 23.3 kcal mol^–1^ in Δ*E*_el_ due to the combination of decreased positive charge at the pnictogen center and increased covalent radius (0.09 Å).^30^ Ultimately, these large changes in Δ*E*_el_ are reflected in Δ*E* due to the increased prominence of electrostatic contributions in these cationic species.

Despite the apparent increased importance of Δ*E*_el_ in determining Δ*E*, SbPh_4_^+^—even with its lower Δ*E*_el_—is still 16.6 kcal mol^–1^ more acidic than PPh_4_^+^. While the stabilizing interactions (Δ*E*_el_ + Δ*E*_oi_) decrease by 75.3 kcal mol^–1^, they are matched by a 77.3 kcal mol^–1^ decrease in Δ*E*_Pauli_. The 14.7 kcal mol^–1^ decrease in Δ*E*_strain_ then drives the increased Lewis acidity of SbPh_4_^+^.

Finally, we analyzed the neutral PnPh_3_Cat series. Oxidation of pnictogens using *ortho*-chloranil has been repeatedly applied to produce active anion receptors and Lewis acid catalysts.^3f,13^ Due to the differing substituents, two isomers are possible upon binding F^–^: one where F is *trans* to Ph and the other with F *trans* to Cat. Because the same trends hold in both series (Supporting Information) and the isomer with F *trans* to Ph is 1.5 kcal mol^–1^ lower in energy for Sb, we have focused our analysis on this series. Overall, these Δ*E* values are lower than their PnF_5_ and PnPh_4_^+^ counterparts yet still higher than those seen for the pnictogen trifluorides. This decreased Lewis acidity is expected due to a reduced σ-hole and a higher-lying σ*-orbital resulting from decreased bond polarity. This reduced polarity produces a less ionic interaction as seen in the relative contributions of Δ*E*_el_ and Δ*E*_oi_: Δ*E*_oi_ contributes 39–46% to the stabilization energy for all pnictogens, whereas it contributes 29–41% in the PnF_5_ and PnPh_4_^+^ series (Chart 2). While the overall Δ*E* values are lower in the PnPh_3_Cat series, it is noteworthy that Δ*E*_strain_ is the lowest among the pentavalent pnictogen series presented in Table 1, indicating the benefits of preorganization that the catecholate provides.^23^ As also seen in the PnF_5_ and PnPh_4_^+^ series, Sb has the greatest Lewis acidity despite having the lowest magnitude of stabilizing contributions due to such a significant reduction in destabilizing contributions.

## Conclusions

Though FIAs provide a way to compare the strengths of Lewis acids, activation strain analysis paired with EDA allows deeper insight into the underlying contributions to Lewis acid strength. We have confirmed that oxidation from Pn^III^ to Pn^V^ produces an increase in Δ*E*_el_ and Δ*E*_oi_ due to a deeper σ-hole and a lower-energy σ*-orbital. While it was already known that Sb-based acids are strong Lewis acids, our analysis highlights the significance of increased molecular flexibility and decreased Pauli repulsion in the preeminence of Sb among the pentavalent pnictogens. Despite lower stabilizing contributions from Δ*E*_el_ and Δ*E*_oi_ moving down the group, Sb exhibits greater Lewis acidity due to lower destabilizing contributions from Δ*E*_strain_ and Δ*E*_Pauli_. The decrease in Δ*E*_Pauli_ prevents drastic changes in Δ*E*_int_ by offsetting the decreases in Δ*E*_el_ and Δ*E*_oi_, thereby allowing the significant reduction in Δ*E*_strain_ to drive the dramatic increase in Δ*E* from P to Sb. Additionally, we not only confirmed the importance of electrostatic contributions for cationic Lewis acids but also demonstrated that the pnictogen bond has substantial orbital contribution. Our hope is that this work informs future applications of pnictogen-based Lewis acids.

## Supporting Information Available

Complete data table; bar graphs; and optimized structures in *XYZ* format (PDF)

## Notes

A draft of this work has been deposited to ChemRxiv.^33^
