# Supplementary material for: Analyzing Fluoride Binding by Group 15 Lewis Acids: Pnictogen Bonding in the Pentavalent State
Source: Inorg Chem. 2023 Aug 8;62(33):13566–72. doi: 10.1021/acs.inorgchem.3c01987 (PMC10862541; doi:10.1021/acs.inorgchem.3c01987)
Supplement: Supplementary file 1 — ic3c01987_si_001.pdf [file ic3c01987_si_001.pdf]

Analyzing Fluoride Binding by Group 15 Lewis Acids: Pnictogen Bonding in the Pentavalent State

Logan T. Maltz and François P. Gabbaï*

Department of Chemistry, Texas A&M University, College Station, Texas 77843-3255, United States

SUPPORTING INFORMATION

This PDF file includes

Contents

Full Data Table S2

Energy Decomposition Bar Graphs..S3

XYZ Coordinates of Optimized Geometries S8

Full Data Table

Table S1. Activation Strain and Energy Decomposition Analyses (in kcal mol^{-1}) at Optimized Geometries

Acid	ΔE	ΔE_{strain}	ΔE_{int}	ΔE_{disp}	ΔE_{el}	ΔE_{oi}	ΔE_{Pauli}	$d_{Pn\cdots F}$ (Å)	Charge^a	E_{LUMO} (eV)^b
					$F^-\cdots PnF_3$					
PF_3	-49.6	18.5	-68.1	0.0	-173.2	-125.6	230.7	1.738^c	1.77	-2.16
AsF_3	-60.1	10.2	-70.4	0.0	-161.9	-95.3	186.8	1.847^c	1.84	-2.50
SbF_3	-71.2	9.0	-80.2	0.0	-161.0	-81.5	162.2	1.989^c	1.98	-3.03
BiF_3	-72.9	5.8	-78.7	0.0	-142.9	-62.9	127.1	2.119^c	2.00	-2.94
					$F^-\cdots PnF_5$					
PF_5	-91.6	51.8	-143.4	0.0	-220.1	-154.1	230.9	1.634	2.71	-5.49
AsF_5	-104.5	33.0	-137.5	0.0	-215.1	-129.8	207.5	1.738	2.76	-6.37
SbF_5	-120.3	23.7	-144.1	-0.1	-209.3	-101.4	166.7	1.900	2.96	-6.52
BiF_5	-116.9	17.3	-134.1	-0.1	-194.2	-92.4	152.6	1.997	2.80	-7.34
					$F^-\cdots PnCl_5$					
PCl_5	-97.1	30.9	-128.0	0.0	-228.6	-201.0	301.6	1.618	0.93	-5.66
$AsCl_5$	-94.6	22.0	-116.6	-0.1	-212.1	-156.0	251.6	1.737	1.12	-6.09
$SbCl_5$	-106.1	18.3	-124.4	-0.1	-200.9	-120.9	197.4	1.904	1.60	-5.90
$BiCl_5$	-102.2	14.6	-116.9	-0.1	-184.1	-105.3	172.6	2.004	1.55	-6.68
					$F^-\cdots PnBr_5$					
PBr_5	-97.9	24.2	-122.2	0.0	-228.0	-209.8	315.7	1.620	0.52	-5.56
$AsBr_5$	-91.9	17.9	-109.7	-0.1	-209.1	-160.5	259.9	1.741	0.70	-5.87
$SbBr_5$	-101.6	15.5	-117.1	-0.1	-195.9	-124.9	203.7	1.910	1.21	-5.62
$BiBr_5$	-97.0	12.7	-109.7	-0.1	-179.3	-107.5	177.3	2.011	1.21	-6.27
					$F^-\cdots PnPh_4^+$					
PPh_4^+	-125.4	33.3	-158.7	-0.5	-251.1	-155.8	248.7	1.724	1.52	-5.37
$AsPh_4^+$	-123.4	23.7	-147.1	-0.5	-230.8	-118.9	203.2	1.852	1.64	-5.32
$SbPh_4^+$	-142.0	18.6	-160.6	-0.4	-229.4	-102.2	171.4	2.000	1.94	-5.81
$BiPh_4^+$	-138.9	14.2	-153.0	-0.4	-206.1	-84.9	138.3	2.132	1.78	-6.01
					$F^-\cdots PnPh_3Cat$ (F^- *trans* to Ph)					
PPh_3Cat	-75.0	31.3	-106.3	-0.5	-199.4	-170.2	263.8	1.683	1.76	-2.94
$AsPh_3Cat$	-73.2	20.6	-93.8	-0.5	-180.5	-135.3	222.5	1.801	1.89	-3.13
$SbPh_3Cat$	-85.9	16.0	-101.8	-0.5	-172.4	-112.9	184.0	1.955	2.23	-3.30
$BiPh_3Cat$	-78.8	12.8	-91.6	-0.5	-152.1	-98.7	159.6	2.065	2.05	-3.69
					$F^-\cdots PnPh_3Cat$ (F^- *trans* to Cat)					
PPh_3Cat	-78.3	46.4	-124.6	-0.5	-207.6	-174.3	257.8	1.659	1.87	-3.48
$AsPh_3Cat$	-74.4	35.3	-109.7	-0.5	-189.4	-136.0	216.2	1.787	1.99	-3.57
$SbPh_3Cat$	-84.4	26.1	-110.5	-0.5	-178.9	-111.2	180.0	1.950	2.30	-3.63
$BiPh_3Cat$	-81.9	17.3	-99.2	-0.5	-154.8	-91.0	147.2	2.075	2.11	-3.72

^{a}NPA charge in strained acid without F^-. ^{b}LUMO energy in strained acid without F^-. ^{c}Smaller of two Pn···F distances.

Energy Decomposition Bar Graphs

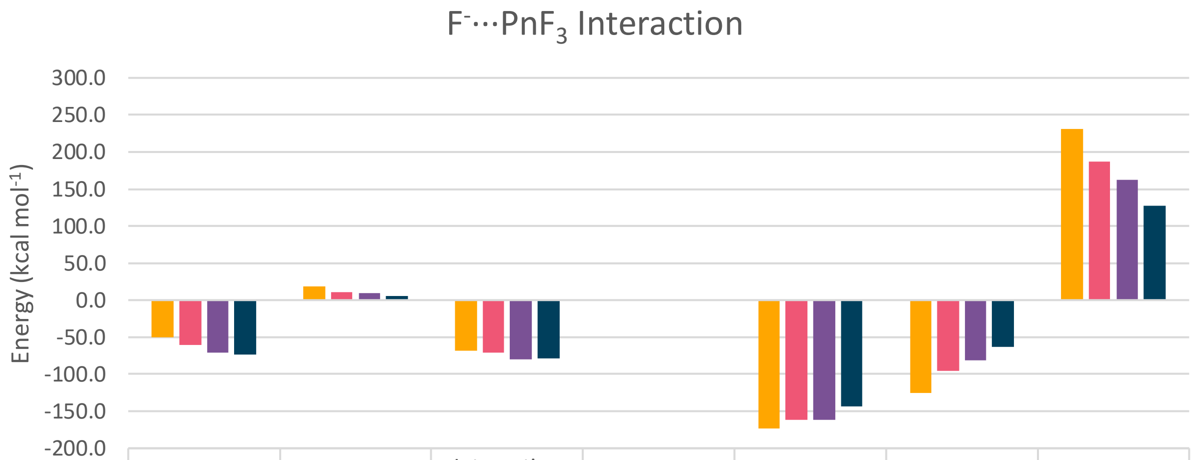

	Total Energy	Strain Energy	Interaction Energy	Dispersion	Electrostatics	Orbital	Pauli
PF3	-49.6	18.5	-68.1	0.0	-173.2	-125.6	230.7
AsF3	-60.1	10.2	-70.4	0.0	-161.9	-95.3	186.8
SbF3	-71.2	9.0	-80.2	0.0	-161.0	-81.5	162.2
BiF3	-72.9	5.8	-78.7	0.0	-142.9	-62.9	127.1

Graph S1. Bar graph of $F^{-}\cdots PnF_3$ interaction

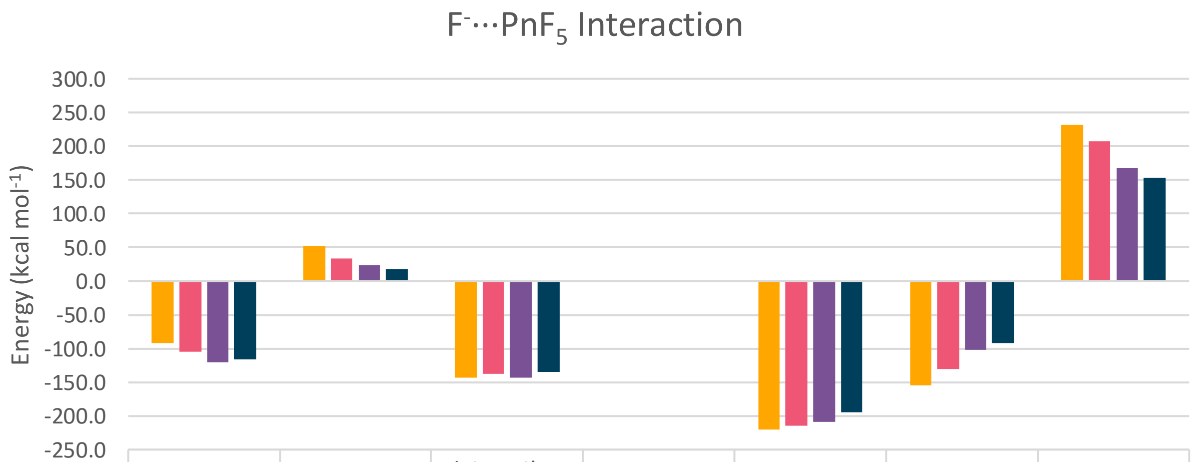

	Total Energy	Strain Energy	Interaction Energy	Dispersion	Electrostatics	Orbital	Pauli
PF5	-91.6	51.8	-143.4	0.0	-220.1	-154.1	230.9
AsF5	-104.5	33.0	-137.5	0.0	-215.1	-129.8	207.5
SbF5	-120.3	23.7	-144.1	-0.1	-209.3	-101.4	166.7
BiF5	-116.9	17.3	-134.1	-0.1	-194.2	-92.4	152.6

Graph S2. Bar graph of $F^{-}\cdots PnF_5$ interaction

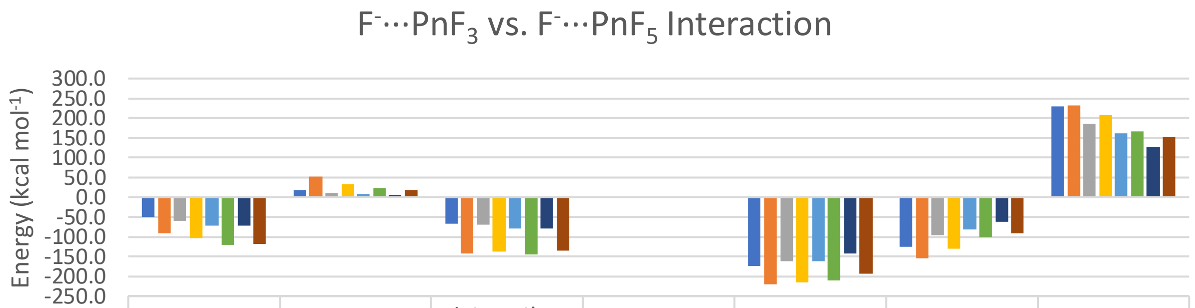

	Total Energy	Strain Energy	Interaction Energy	Dispersion	Electrostatics	Orbital	Pauli
PF3	-49.6	18.5	-68.1	0.0	-173.2	-125.6	230.7
PF5	-91.6	51.8	-143.4	0.0	-220.1	-154.1	230.9
AsF3	-60.1	10.2	-70.4	0.0	-161.9	-95.3	186.8
AsF5	-104.5	33.0	-137.5	0.0	-215.1	-129.8	207.5
SbF3	-71.2	9.0	-80.2	0.0	-161.0	-81.5	162.2
SbF5	-120.3	23.7	-144.1	-0.1	-209.3	-101.4	166.7
BiF3	-72.9	5.8	-78.7	0.0	-142.9	-62.9	127.1
BiF5	-116.9	17.3	-134.1	-0.1	-194.2	-92.4	152.6

Graph S3. Bar graph of $F^-\cdots PnF_3$ vs. $F^-\cdots PnF_5$ interaction

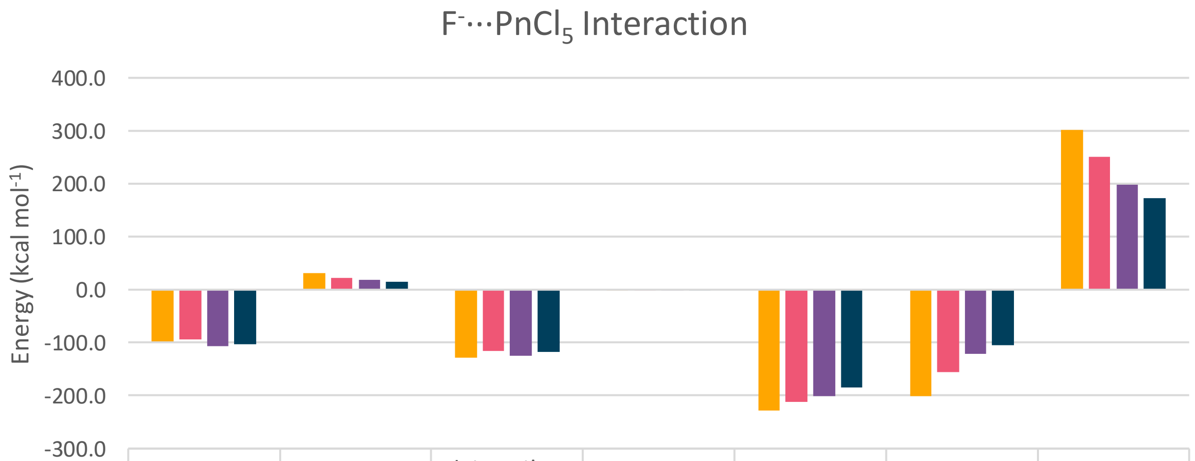

	Total Energy	Strain Energy	Interaction Energy	Dispersion	Electrostatics	Orbital	Pauli
PCl5	-97.1	30.9	-128.0	0.0	-228.6	-201.0	301.6
AsCl5	-94.6	22.0	-116.6	-0.1	-212.1	-156.0	251.6
SbCl5	-106.1	18.3	-124.4	-0.1	-200.9	-120.9	197.4
BiCl5	-102.2	14.6	-116.9	-0.1	-184.1	-105.3	172.6

Graph S4. Bar graph of $F^-\cdots PnCl_5$

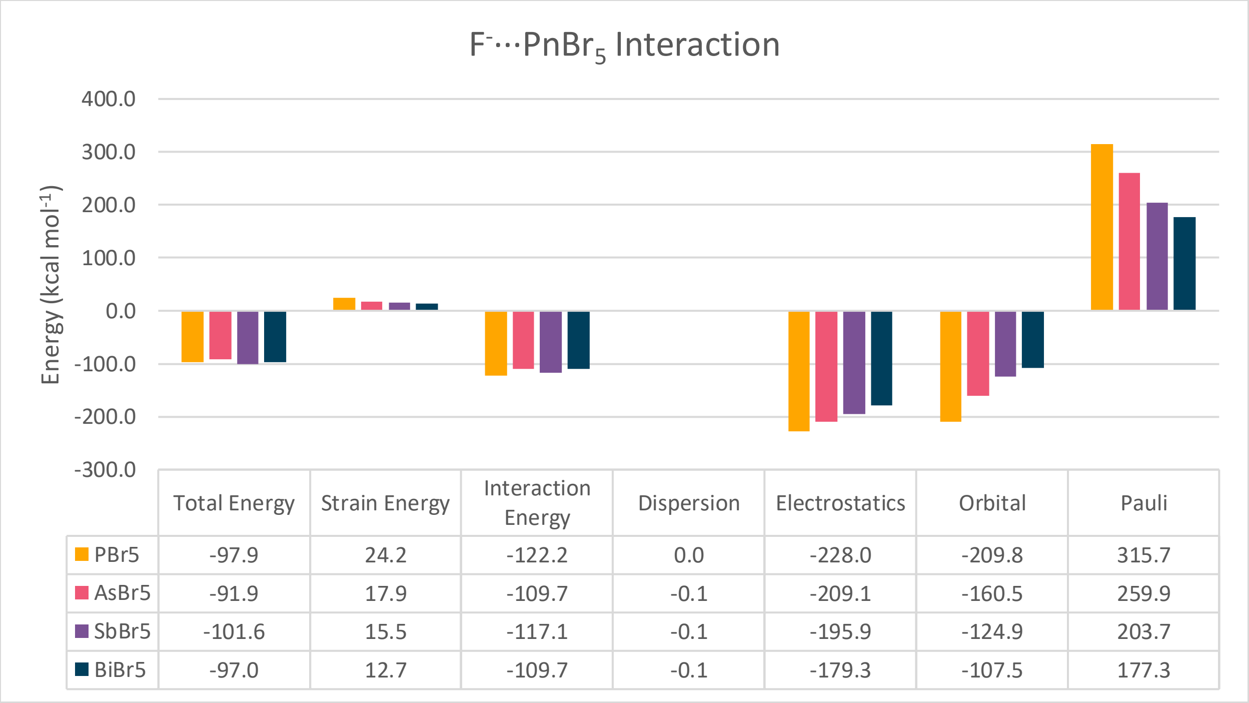

	Total Energy	Strain Energy	Interaction Energy	Dispersion	Electrostatics	Orbital	Pauli
PBr5	-97.9	24.2	-122.2	0.0	-228.0	-209.8	315.7
AsBr5	-91.9	17.9	-109.7	-0.1	-209.1	-160.5	259.9
SbBr5	-101.6	15.5	-117.1	-0.1	-195.9	-124.9	203.7
BiBr5	-97.0	12.7	-109.7	-0.1	-179.3	-107.5	177.3

Graph S5. Bar graph of $F^{-}\cdots PnBr_5$

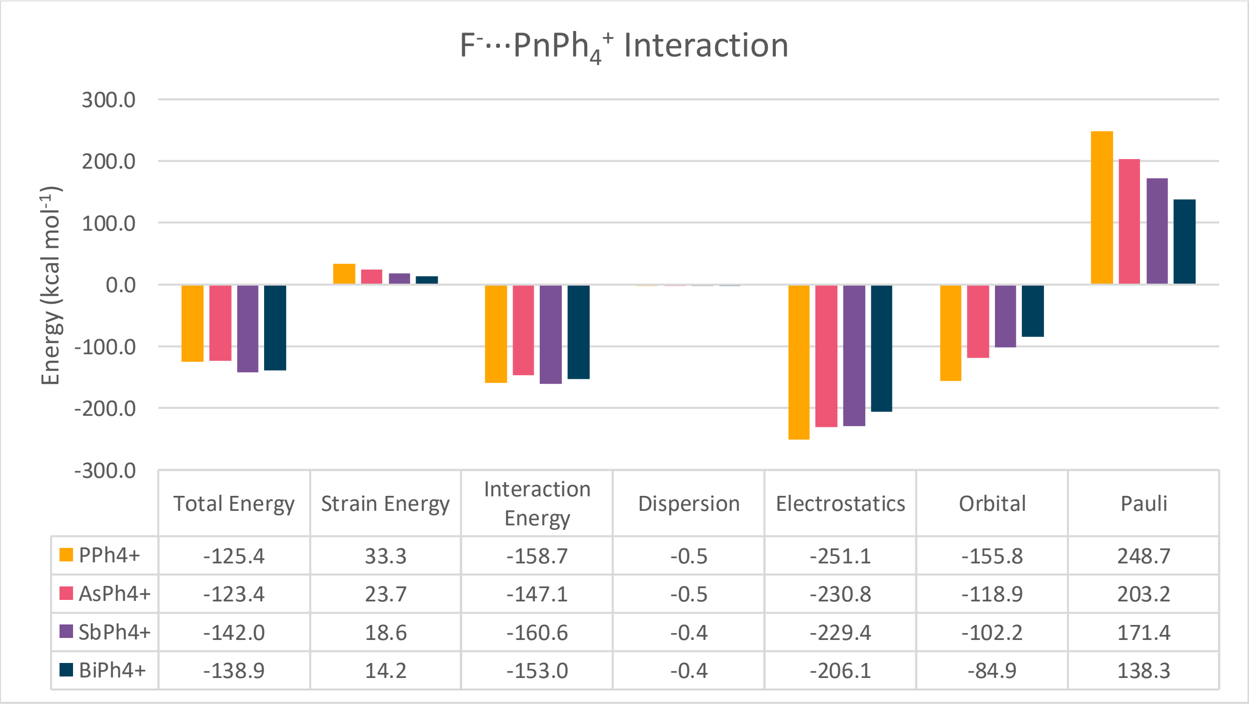

	Total Energy	Strain Energy	Interaction Energy	Dispersion	Electrostatics	Orbital	Pauli
PPh4+	-125.4	33.3	-158.7	-0.5	-251.1	-155.8	248.7
AsPh4+	-123.4	23.7	-147.1	-0.5	-230.8	-118.9	203.2
SbPh4+	-142.0	18.6	-160.6	-0.4	-229.4	-102.2	171.4
BiPh4+	-138.9	14.2	-153.0	-0.4	-206.1	-84.9	138.3

Graph S6. Bar graph of $F^{-}\cdots PnPh_4^{+}$

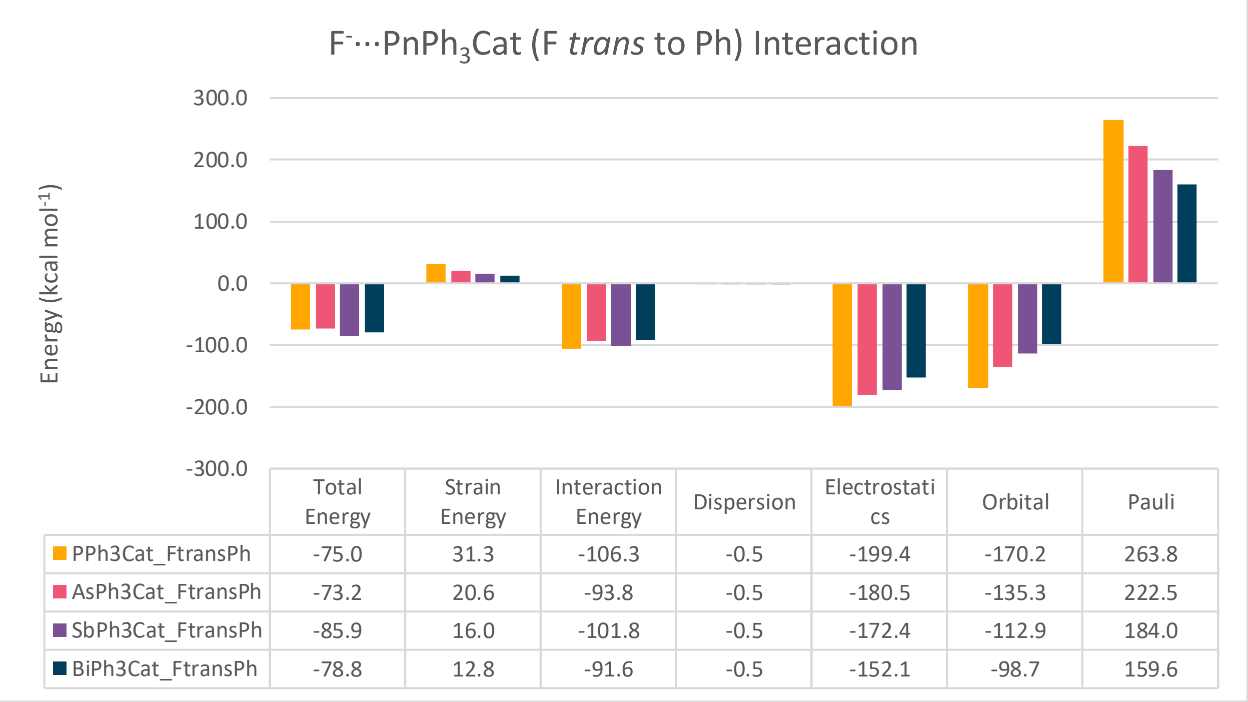

	Total Energy	Strain Energy	Interaction Energy	Dispersion	Electrostatics	Orbital	Pauli
PPh3Cat_FtransPh	-75.0	31.3	-106.3	-0.5	-199.4	-170.2	263.8
AsPh3Cat_FtransPh	-73.2	20.6	-93.8	-0.5	-180.5	-135.3	222.5
SbPh3Cat_FtransPh	-85.9	16.0	-101.8	-0.5	-172.4	-112.9	184.0
BiPh3Cat_FtransPh	-78.8	12.8	-91.6	-0.5	-152.1	-98.7	159.6

Graph S7. Bar graph of $F^{-}\cdots PnPh_3Cat$ (F *trans* to Ph) interaction

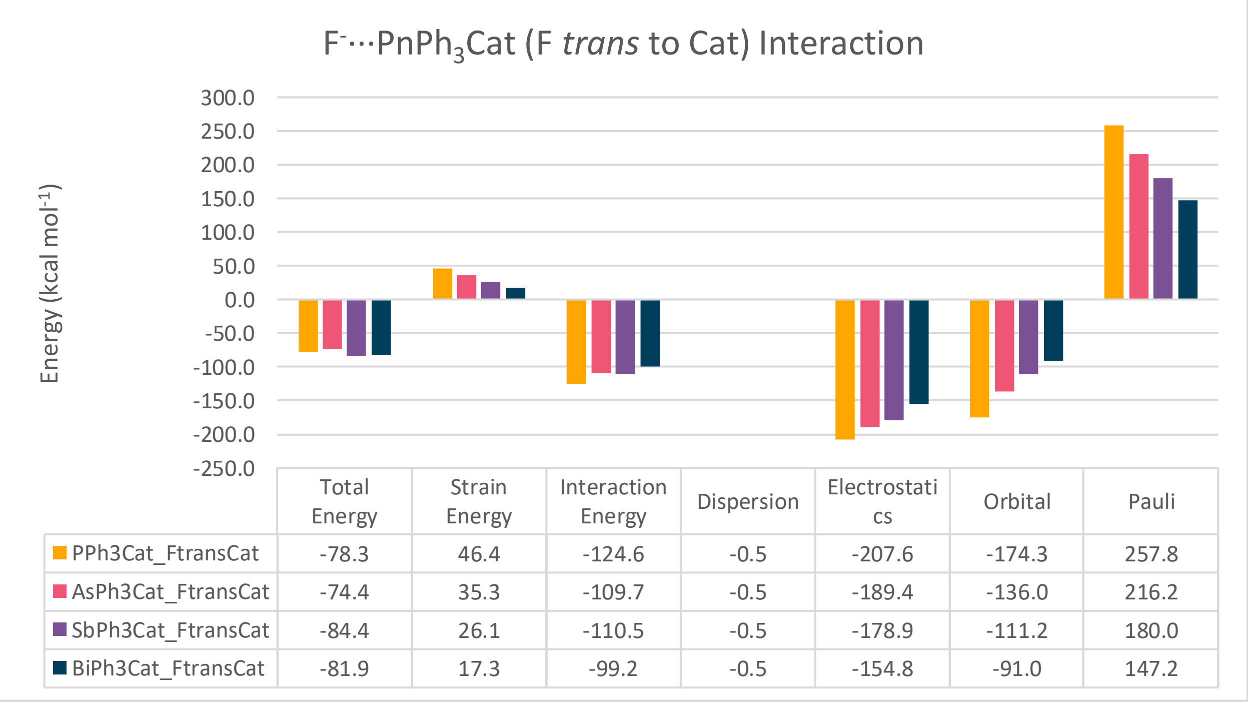

	Total Energy	Strain Energy	Interaction Energy	Dispersion	Electrostatics	Orbital	Pauli
PPh3Cat_FtransCat	-78.3	46.4	-124.6	-0.5	-207.6	-174.3	257.8
AsPh3Cat_FtransCat	-74.4	35.3	-109.7	-0.5	-189.4	-136.0	216.2
SbPh3Cat_FtransCat	-84.4	26.1	-110.5	-0.5	-178.9	-111.2	180.0
BiPh3Cat_FtransCat	-81.9	17.3	-99.2	-0.5	-154.8	-91.0	147.2

Graph S8. Bar graph of $F^{-}\cdots PnPh_3Cat$ (F *trans* to Cat) interaction

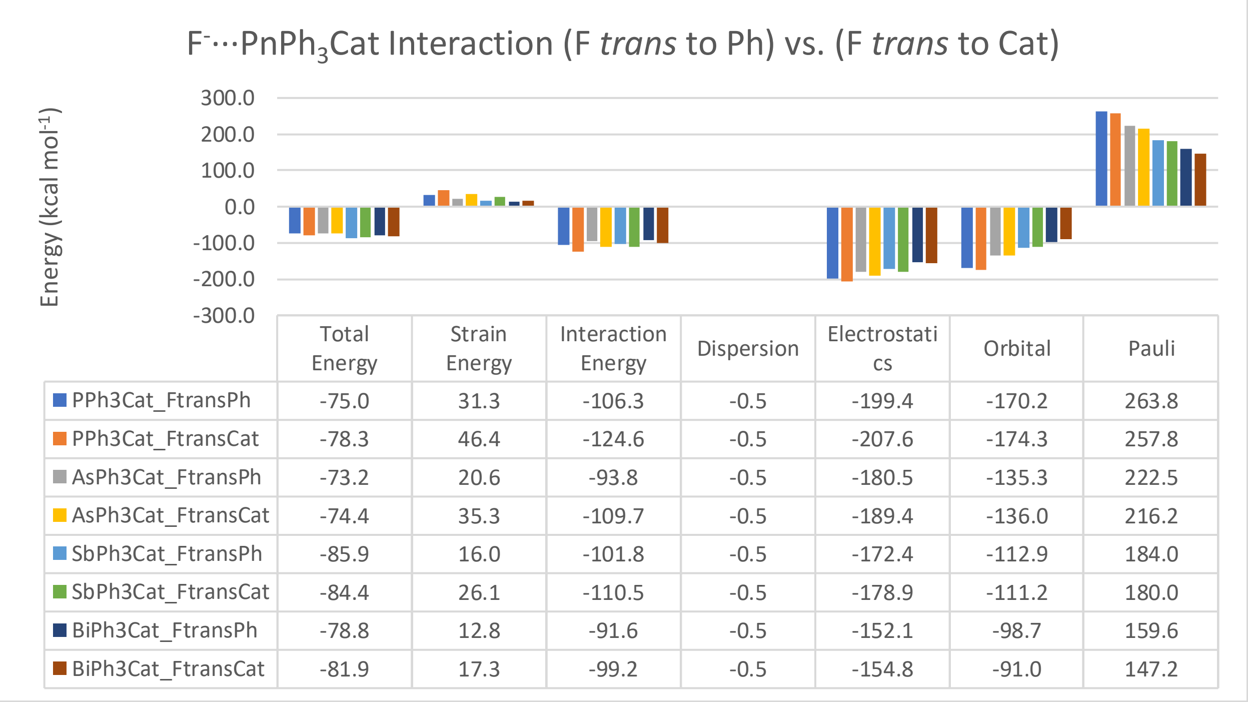

	Total Energy	Strain Energy	Interaction Energy	Dispersion	Electrostatics	Orbital	Pauli
PPh3Cat_FtransPh	-75.0	31.3	-106.3	-0.5	-199.4	-170.2	263.8
PPh3Cat_FtransCat	-78.3	46.4	-124.6	-0.5	-207.6	-174.3	257.8
AsPh3Cat_FtransPh	-73.2	20.6	-93.8	-0.5	-180.5	-135.3	222.5
AsPh3Cat_FtransCat	-74.4	35.3	-109.7	-0.5	-189.4	-136.0	216.2
SbPh3Cat_FtransPh	-85.9	16.0	-101.8	-0.5	-172.4	-112.9	184.0
SbPh3Cat_FtransCat	-84.4	26.1	-110.5	-0.5	-178.9	-111.2	180.0
BiPh3Cat_FtransPh	-78.8	12.8	-91.6	-0.5	-152.1	-98.7	159.6
BiPh3Cat_FtransCat	-81.9	17.3	-99.2	-0.5	-154.8	-91.0	147.2

Graph S9. Bar graph of $F^{-}\cdots PnPh_3Cat$ (F *trans* to Ph) vs. (F *trans* to Cat) interaction

XYZ Coordinates of Optimized Geometries

Table S2. XYZ coordinates of the optimized geometries of PnF_3 and $F^{-}\cdots PnF_3$

		**PF_3**	
P	0.00129099441724	0.00000118572170	0.60384033935086
F	-0.69527993056828	1.20060077709404	-0.18079210628771
F	-0.69527528589358	-1.20059897891080	-0.18079263790792
F	1.38926422204462	-0.00000298390493	-0.18006259515523
		**$F^{-}\cdots PF_3$**	
P	0.48457731287740	-0.04619422044033	-0.00000275616378
F	-0.58861255447596	-0.00361133439584	-1.24236881320764
F	-0.58861098211151	-0.00361210993429	1.24236641223664
F	0.26825914129172	-1.77136231118134	0.00000281939036
F	0.40276508241836	1.68970897595179	0.00000233774442
		**AsF_3**	
As	0.00015234397849	-0.00001380837142	0.66222304237706
F	-0.73911923080124	1.27999012433484	-0.20246343410982
F	-0.73915690417673	-1.28000268099614	-0.20245348111071
F	1.47812279099948	0.00002636503271	-0.20237212715654
		**$F^{-}\cdots AsF_3$**	
As	0.53217460172878	0.14296521295646	0.00000331931670
F	0.09552687192483	1.93849738368833	-0.00000215570109
F	-0.58841114735350	-0.00218785540480	1.34025787296843
F	-0.58840265082868	-0.00218632555526	-1.34026190035753
F	0.55717132452856	-1.70415041568473	0.00000286377350
		**SbF_3**	
Sb	-0.00372321853577	-0.00000178239726	0.69562345811536
F	-0.80537544342781	1.40843591074175	-0.27653896114025
F	-0.80538087893374	-1.40843723164639	-0.27653553457397
F	1.61447954089733	0.00000310330190	-0.27885796240114
		**$F^{-}\cdots SbF_3$**	
Sb	0.60215443030031	0.18343617854963	-0.00001222790002
F	-0.05516990364990	2.06034145235068	0.00000320071366
F	-0.55759349660945	0.01881891066084	1.53232771457663
F	-0.55768054680935	0.01878374307145	-1.53228150265170
F	0.52783351676839	-1.80957928463259	-0.00003718473857
		**BiF_3**	
Bi	-0.00429657488197	-0.00004122599365	0.72153843262038
F	-0.85151412211108	1.49082144778256	-0.28507704902422
F	-0.85167229425317	-1.49087615866543	-0.28500813618708
F	1.70748299124622	0.00009593687651	-0.28776224740908
		**$F^{-}\cdots BiF_3$**	
Bi	0.57612348045270	0.18347174758250	0.00010272336195
F	0.09146225483070	2.24730653547466	-0.00005519410119
F	-0.70942548443278	-0.01393913887803	1.54680487076245
F	-0.70931608358020	-0.01415548547661	-1.54664230548915
F	0.71069983272958	-1.93088265870252	-0.00021009453406

Table S3. XYZ coordinates of the optimized geometries of PX_5 and $F^{-}\cdots PX_5$

		**PF_5**	
P	1.42663887440498	-0.90476074032687	-0.00000039893666
F	1.41772827916285	-0.90476692024430	1.59197692559463
F	0.65842999037402	0.46186943505723	0.00000341370261
F	0.65843281861119	-2.27138670854287	0.00000194045459
F	1.41772116887907	-0.90476830720182	-1.59197802829646
F	2.99247802856788	-0.90475809874138	-0.00000385251871
		**$F^{-}\cdots PF_5$**	
P	0.78618592336219	0.10922302948850	-0.43796289451433
F	0.78618627208429	-1.52514678429268	-0.43796320623958
F	-0.84818445917587	0.10922290487310	-0.43796252470802
F	0.78618604130206	0.10922262798939	-2.07233325400604
F	2.42055627387329	0.10922258888456	-0.43796309384589
F	0.78618595600046	1.74359331644287	-0.43796372255263
F	0.78618592255356	0.10922289661425	1.19640755586649
		**PCl_5**	
P	1.43021511331430	-0.90476185873668	-0.00000009652209
Cl	1.43407008703956	-0.90476220461524	2.12557460122581
Cl	0.40577667664999	0.84791776134772	0.00000015232013
Cl	0.40577687175684	-2.65744145467246	0.00000049099057
Cl	1.43407019618269	-0.90476209783448	-2.12557525751508
Cl	3.46152021505661	-0.90476148548886	0.00000010950066
		**$F^{-}\cdots PCl_5$**	
P	0.78618599626684	0.10922285607519	-0.37375585911426
Cl	0.78618570669689	-2.04172778657674	-0.35472515063633
Cl	-1.36476465809129	0.10922188709453	-0.35472579462499
Cl	0.78618591801185	0.10922373528079	-2.51770448597875
Cl	2.93713682920443	0.10922203837348	-0.35472573331433
Cl	0.78618585797507	2.26017375820538	-0.35472495440049
F	0.78618627993618	0.10922409154735	1.24462083806917
		**PBr_5**	
P	1.42987334233928	-0.90476167272297	-0.00000073765692
Br	1.43433151535880	-0.90475726649502	2.33910123747740
Br	0.31742797765890	1.00207197989842	-0.00000130224920
Br	0.31743528354454	-2.81159936196562	0.00000114997207
Br	1.43433055002548	-0.90475537890306	-2.33910119258093
Br	3.63803049107300	-0.90476963981176	0.00000084503758
		**$F^{-}\cdots PBr_5$**	
P	0.78618527560387	0.10922318552711	-0.34827376948471
Br	0.78618649480607	-2.23975573674192	-0.32334703704908
Br	-1.56279628571825	0.10922079732991	-0.32334750411045
Br	0.78618707072253	0.10922499852540	-2.69597822138366
Br	3.13516765135014	0.10922055608330	-0.32334921625279
Br	0.78618625346656	2.45820249169380	-0.32334822469897
F	0.78618546976907	0.10922428758239	1.27190283297967

Table S4. XYZ coordinates of the optimized geometries of AsX_5 and $F^-\cdots AsX_5$

AsF_5

As	1.42445604071886	-0.90476076884398	0.00000071160198
F	1.40992007196023	-0.90476116320430	1.69938412302489
F	0.61129739280001	0.56906749408965	0.00000131521106
F	0.61130360998671	-2.37859222862120	0.00000002073304
F	1.40991011625625	-0.90476240419420	-1.69938223722324
F	3.10454192827792	-0.90476226922598	-0.00000393334772

$F^-\cdots AsF_5$

As	0.78618583650669	0.10922275041536	-0.43796240311215
F	0.78618646371744	-1.62924589758054	-0.43796354319406
F	-0.95228230415038	0.10922259037704	-0.43796371103517
F	0.78618476777103	0.10922285930471	-2.17643067421809
F	2.52465400054075	0.10922291803083	-0.43796258350868
F	0.78618679126813	1.84769168183031	-0.43796355985221
F	0.78618637434631	0.10922367762227	1.30050533492037

$AsCl_5$

As	1.43129278572986	-0.90475937858716	0.00000317093034
Cl	1.44141176770687	-0.90476390846608	2.20568285250881
Cl	0.34041153932461	0.93914863137863	-0.00000184891635
Cl	0.34041363133151	-2.74867067320900	-0.00000148363641
Cl	1.44141137959110	-0.90476379016741	-2.20568124659418
Cl	3.57648805631603	-0.90476222094899	-0.00000144429221

$F^-\cdots AsCl_5$

As	0.78618497102080	0.10922135534855	-0.38002430496655
Cl	0.78618676771840	-2.12639862506476	-0.35502787041181
Cl	-1.44943525585493	0.10922437569885	-0.35502806274128
Cl	0.78618612413970	0.10922312528318	-2.62216980934902
Cl	3.02180650892575	0.10922430231692	-0.35502835345152
Cl	0.78618669433236	2.34484348877776	-0.35502828309950
F	0.78618611971790	0.10922255763949	1.35656554401968

$AsBr_5$

As	1.43205221739703	-0.90476274424775	0.00000303055804
Br	1.44504438697627	-0.90475130994894	2.40508760631870
Br	0.25133134530817	1.08231242191454	-0.00000270133866
Br	0.25134391597143	-2.89184375548690	-0.00000046403380
Br	1.44504311558215	-0.90474962666026	-2.40508704893317
Br	3.74661417876494	-0.90477632557068	-0.00000042257110

$F^-\cdots AsBr_5$

As	0.78618788465635	0.10922161722227	-0.35542018026224
Br	0.78617986867064	-2.31644538361236	-0.32505014607566
Br	-1.63948081450785	0.10922317475397	-0.32504520427838
Br	0.78619458324248	0.10922623484053	-2.79548373638645
Br	3.21185151587282	0.10922162190442	-0.32505090011679
Br	0.78617831584554	2.53489017521823	-0.32505321979901
F	0.78619057622003	0.10922313967293	1.38536224691853

Table S5. XYZ coordinates of the optimized geometries of SbX_5 and $F^{-}\cdots SbX_5$

		**SbF_5**	
Sb	1.41372620665303	-0.90479539912986	-0.00000219180003
F	1.34452670438983	-0.90481752150911	1.86368977705243
F	0.60178619303405	0.76818863237335	-0.00000030019269
F	0.60190306878065	-2.57753874686088	-0.00000344503161
F	1.34451787964585	-0.90482045620636	-1.86367576522974
F	3.26496910749658	-0.90478784866714	-0.00000807479836
		**$F^{-}\cdots SbF_5$**	
Sb	0.78618584671168	0.10922307339877	-0.43791783041300
F	0.78618418019346	-1.79050129537240	-0.43796313082674
F	-1.11352842745781	0.10922404393207	-0.43795894073211
F	0.78618486967759	0.10922397643521	-2.33765493603092
F	2.68590572326880	0.10922488140837	-0.43795961382364
F	0.78618501755979	2.00894177732802	-0.43796389680998
F	0.78618472004646	0.10922412286994	1.46167720863639
		**$SbCl_5$**	
Sb	1.43268931481888	-0.90476224686000	-0.00000010675561
Cl	1.44961402054102	-0.90476237902930	2.34676544140084
Cl	0.24396707757832	1.08284252108109	0.00000021595279
Cl	0.24396502285962	-2.89236689840557	0.00000029615639
Cl	1.44961576715993	-0.90476251160260	-2.34676418660391
Cl	3.75157795704222	-0.90475982518362	-0.00000166015050
		**$F^{-}\cdots SbCl_5$**	
Sb	0.78618587102206	0.10922296829149	-0.38565889998508
Cl	0.78618530901978	-2.26866958715090	-0.35661791982934
Cl	-1.59170866412850	0.10922160647347	-0.35662084771820
Cl	0.78618794099522	0.10922332755693	-2.77150066898756
Cl	3.16407956683629	0.10922097498749	-0.35662216076043
Cl	0.78618467753215	2.48711649173795	-0.35661683764913
F	0.78618722872299	0.10922479810356	1.51789619492974
		**$SbBr_5$**	
Sb	1.43751647048598	-0.90476215327562	-0.00000289060464
Br	1.47019123949320	-0.90475715934194	2.52427230633977
Br	0.13614660480000	1.20367268118832	0.00000407200166
Br	0.13615610116336	-3.01319837502555	0.00000581331369
Br	1.47019619535599	-0.90475589954420	-2.52427933594309
Br	3.92122254870146	-0.90477043400101	0.00000003489262
		**$F^{-}\cdots SbBr_5$**	
Sb	0.78618668559537	0.10922367321217	-0.35978984773643
Br	0.78618665994007	-2.44337640809276	-0.33138964803960
Br	-1.76641420749055	0.10922385936373	-0.33137841126698
Br	0.78618251219952	0.10921871440203	-2.93096682710836
Br	3.33878989259961	0.10922174797980	-0.33137510626657
Br	0.78618454855337	2.66182386083558	-0.33138374781049
F	0.78618583860261	0.10922513229944	1.55054244822843

Table S6. XYZ coordinates of the optimized geometries of BiX_5 and $F^{-}\cdots BiX_5$

		**BiF_5**	
Bi	1.41047747357827	-0.90476940564478	0.00000267447925
F	1.33480189929044	-0.90475442737025	1.96084493722586
F	0.56510693848334	0.86233522211623	0.00000996477444
F	0.56515516527306	-2.67189956206038	0.00001192715142
F	1.33473531571885	-0.90475453350818	-1.96083846731073
F	3.36115236765603	-0.90472863353263	-0.00003103632024
		**$F^{-}\cdots BiF_5$**	
Bi	0.78612203735485	0.10920181700816	-0.43794763796877
F	0.78613289968926	-1.88750554581199	-0.43787696310028
F	-1.21117311193616	0.10920215008559	-0.43802227814116
F	0.78611583945745	0.10919642889421	-2.43515764179328
F	2.78387757125028	0.10920071429823	-0.43804282003703
F	0.78613146414332	2.10607474617834	-0.43788377195482
F	0.78609523004097	0.10919026934744	1.55918997299535
		**$BiCl_5$**	
Bi	1.43337799464835	-0.90476139202195	-0.00000006310413
Cl	1.45278742477191	-0.90476373664061	2.44487952314358
Cl	0.18862911672850	1.16899072594200	0.00000222357904
Cl	0.18861865967797	-2.97851665494705	-0.00000133850966
Cl	1.45278904810738	-0.90476695474213	-2.44487891563301
Cl	3.85522691606587	-0.90475332759027	-0.00000142947582
		**$F^{-}\cdots BiCl_5$**	
Bi	0.78618555288475	0.10922276932209	-0.38709593805397
Cl	0.78618728258847	-2.36639247579266	-0.35390733542283
Cl	-1.68943035190929	0.10922478669298	-0.35390824134996
Cl	0.78618817242855	0.10922366579218	-2.88019045929467
Cl	3.26180078301423	0.10922173404103	-0.35391022038866
Cl	0.78618422993039	2.58483766838279	-0.35390882340285
F	0.78618626106290	0.10922243156158	1.61717987791294
		**$BiBr_5$**	
Bi	1.44149634845089	-0.90476350867005	-0.00000080860832
Br	1.48291656288938	-0.90474472044601	2.61589698163335
Br	0.06832993178880	1.27752928837528	0.00000316770323
Br	0.06832986793258	-3.08707141580816	-0.00000130142122
Br	1.48291674796868	-0.90474848616544	-2.61589905550351
Br	4.02743970096967	-0.90477249728560	0.00000101619647
		**$F^{-}\cdots BiBr_5$**	
Bi	0.78618570725929	0.10922223779441	-0.35702172965328
Br	0.78618942855809	-2.53652135175881	-0.33165197768742
Br	-1.85955685961276	0.10922063875877	-0.33164287613968
Br	0.78617870484507	0.10922382709193	-3.03610923951076
Br	3.43192933059302	0.10922426627266	-0.33163778189944
Br	0.78619305623289	2.75496966356190	-0.33165393327911
F	0.78618256212439	0.10922129827914	1.65397639816968

Table S7. XYZ coordinates of the optimized geometries of PPh_4^+

PPh_4^+

P	0.89294693826650	-0.39757799381801	0.00281590346932
C	1.62433265784658	0.39097214454636	1.43985106262458
C	1.65833384125029	-0.30618720116056	2.64978082888338
C	2.12372290233787	1.68818932106991	1.36288501445186
C	2.18480182569839	0.30248600138365	3.77562267628930
C	2.65146270353169	2.28717121698220	2.49583957531472
C	2.68028570369098	1.59773198893269	3.69857641349785
C	1.45318766730233	-2.10256068368450	-0.02997982895884
C	0.58452032726241	-3.13194953174727	-0.38310122083092
C	2.78861267144214	-2.38042261811623	0.26989404220383
C	1.05584892078019	-4.43366321234731	-0.44724613790009
C	3.24940326418575	-3.68372965468147	0.20066735448938
C	2.38434722761560	-4.70834934556677	-0.16048402806845
C	-0.89806270516551	-0.32479229277280	0.09594493998496
C	-1.64329684914308	-0.57133868584243	-1.05931782547727
C	-1.54100583083619	-0.05017108385327	1.29981141178609
C	-3.02570128686887	-0.55496745319668	-0.99990186449199
C	-2.92627169947345	-0.03171498910696	1.34735361730970
C	-3.66621086578060	-0.28643664008487	0.20281310071254
C	1.39363754244013	0.45287920796350	-1.49637905506858
C	0.74840749913658	1.64235030597291	-1.84155271357030
C	2.43078566651928	-0.03848185388698	-2.28485156004290
C	1.15169022827643	2.33856019769397	-2.96766959668777
C	2.82505376004636	0.66563766256330	-3.41187118219506
C	2.19047945174356	1.85144972198961	-3.75015167453912
H	1.28707609149444	-1.32193634758153	2.71472985839886
H	2.11795173959404	2.22964496447991	0.42622476952788
H	2.21404748539290	-0.23630820319065	4.71267423031201
H	3.04590850883505	3.29198202086409	2.43549588830196
H	3.09627359649081	2.06785228182432	4.57941088733965
H	-0.45734264060880	-2.93130640367244	-0.59434550276279
H	3.46626208914374	-1.58909852946157	0.56685851523837
H	0.38103434131325	-5.23434511630962	-0.71639644366516
H	4.28246016670386	-3.90060118762718	0.43487096338590
H	2.74658882872567	-5.72626377397295	-0.21013070983944
H	-1.15087426990257	-0.76358732671950	-2.00499134060022
H	-0.97360258968548	0.16350546655188	2.19587905907077
H	-3.60346912788365	-0.74408608887076	-1.89406617877075
H	-3.42651087527487	0.18849181374630	2.28022367026127
H	-4.74679768506288	-0.26798604801038	0.24474678427305
H	-0.07162968115057	2.02110320890302	-1.24361901262313
H	2.92288893369251	-0.96975629236542	-2.03739616591924
H	0.65202868343219	3.25863132426881	-3.23765482522866
H	3.62651409791781	0.28293055253902	-4.02839564017453
H	2.50138906472714	2.39560553537272	-4.63164071971206

Table S8. XYZ coordinates of the optimized geometries of $F^{-}\cdots PPh_4^{+}$

	$F^{-}\cdots PPh_4^{+}$		
P	0.43039964422011	0.27044384614292	0.12564449947330
F	0.47917163596598	0.14497023692357	-1.59329112988235
C	-0.48025552548080	-1.33050032429627	0.09779849074646
C	-0.07442850993396	-2.37565404866517	-0.72909050353924
C	-1.60200866605835	-1.51104742411680	0.90327698508901
C	-0.75491459904771	-3.58303771965658	-0.72215254034402
C	-2.31345479146542	-2.70085795604774	0.86911934283277
C	-1.88414840071712	-3.74534139338965	0.06585886358588
C	0.33711552277141	0.33298455695834	2.03671033944909
C	-0.31473648578703	1.31361735811841	2.77655668837822
C	0.94326888094434	-0.71251758884185	2.74050188097410
C	-0.35056756198787	1.26288436730450	4.16812269195306
C	0.91455701917277	-0.76863108331407	4.12173366306069
C	0.26526603078771	0.22594428402513	4.84442713533153
C	-0.49398087542597	1.81881147355230	-0.15009453277067
C	0.13573203431845	3.05021949267649	-0.01389838096720
C	-1.82876453653583	1.77248280100202	-0.53567278475427
C	-0.56882970946817	4.22333317645350	-0.24097960401028
C	-2.53396063646470	2.94713574106317	-0.74811624970628
C	-1.90628319240426	4.17531183182016	-0.60286912024621
C	2.27268692737352	0.37106545443369	0.10807217182185
C	3.02130478937966	-0.22350018018599	-0.90916662812631
C	2.95326031286395	1.07053208534810	1.10512845389024
C	4.40543994895924	-0.15277134782930	-0.90303840069646
C	4.33537719398595	1.17759864675169	1.08563783998764
C	5.06759136604433	0.55336125832328	0.08897098735760
H	0.78336375099627	-2.26553277571070	-1.37536113587569
H	-1.92676979046096	-0.72406829241010	1.57109474550385
H	-0.40991883201271	-4.39365938673493	-1.35044644522280
H	-3.19518814496506	-2.81403840286895	1.48579769151485
H	-2.42554636225867	-4.68222066298323	0.05517156785542
H	-0.80992815528663	2.13759394564821	2.28083605159412
H	1.45222390472797	-1.50157348073905	2.19738829780485
H	-0.86456915752876	2.04120947837826	4.71777486874826
H	1.39608373761463	-1.58896765626622	4.63837112425996
H	0.23954847006970	0.18620127270159	5.92570397336912
H	1.17960743159717	3.10426580780603	0.26889342002595
H	-2.32645789278142	0.82056109442399	-0.67122338786083
H	-0.06761559983939	5.17668827169874	-0.13614685483301
H	-3.57488653406938	2.90082734688577	-1.04014078003406
H	-2.45601089650402	5.09102692238537	-0.77611061637471
H	2.53146701929306	-0.74046075184044	-1.71866845870884
H	2.40977744473453	1.54030604452106	1.91268860545446
H	4.96645208421758	-0.63918465328029	-1.69011664632517
H	4.83729459012305	1.74007597134652	1.86166716433728
H	6.14775991632279	0.61945591248450	0.08300188587877

Table S9. XYZ coordinates of the optimized geometries of $AsPh_4^+$

$AsPh_4^+$			
As	0.89322816778285	-0.39655240255249	-0.00050372966592
C	1.65922064274266	0.43864923800959	1.52393498853267
C	1.71618643804726	-0.26412396085842	2.72695940384712
C	2.13667291971212	1.74263735298333	1.43969962400989
C	2.24985456396379	0.35033103144731	3.84801152736518
C	2.66960996647575	2.34574520749096	2.56837151516795
C	2.72455912936668	1.65243569995719	3.76843310801300
C	1.48865367277296	-2.19930846785789	-0.04131531235736
C	0.61234322116258	-3.21641872511112	-0.40696037934374
C	2.81605982652682	-2.48339085347064	0.27647465357907
C	1.07363548409628	-4.52234397294576	-0.46477971008125
C	3.26491848120745	-3.79262091372386	0.21426045336195
C	2.39573288602865	-4.80867897303250	-0.15873408681913
C	-1.00236363774077	-0.32952752391539	0.09654545778759
C	-1.74784252719061	-0.55809205162338	-1.05928232504832
C	-1.63335311959231	-0.07509558940990	1.30998872791160
C	-3.13126485737839	-0.54111638226256	-0.99067931862216
C	-3.01864710586407	-0.05741384999163	1.36480038665768
C	-3.76453419771493	-0.29270078766132	0.21946041472063
C	1.42728782902144	0.51147877856508	-1.58052567852891
C	0.77214420674402	1.68993985418350	-1.93511462177987
C	2.47770220349670	0.02164712860593	-2.35037024354134
C	1.18148144024307	2.38189880989150	-3.06337677621840
C	2.87671541334013	0.72280745653028	-3.47794541336516
C	2.23318442538627	1.90003882207124	-3.83071832122673
H	1.36103100867371	-1.28558639380150	2.79783671633699
H	2.11065174522929	2.28884096693421	0.50512148384292
H	2.29886112373419	-0.19020802861694	4.78332672862893
H	3.04686639689115	3.35717979160685	2.50726883500159
H	3.14421133023029	2.12640980560986	4.64542998992444
H	-0.42562363094895	-3.00768226222187	-0.63373436057674
H	3.49960972553952	-1.70029306894372	0.58293669472690
H	0.39626204064638	-5.31729207880934	-0.74462196875653
H	4.29267048989302	-4.01946536405150	0.46230032624571
H	2.74939005682121	-5.82980265433584	-0.20357806599534
H	-1.26383358907139	-0.73665545228450	-2.01240486267130
H	-1.06168134126162	0.12172442004535	2.20829682919131
H	-3.71456728179747	-0.71616343026670	-1.88419929610657
H	-3.51453837470687	0.14539090959241	2.30402342599075
H	-4.84484853738448	-0.27576172514770	0.26790713524957
H	-0.05799088892876	2.06816786173431	-1.35020102354290
H	2.97886909913952	-0.90256943335409	-2.09091584600953
H	0.67654464268862	3.29567023784172	-3.34494517668227
H	3.68972172380280	0.34553153300685	-4.08272442274338
H	2.54871878817339	2.44186944014310	-4.71194748641057

Table S10. XYZ coordinates of the optimized geometries of $F^{-}\cdots AsPh_4^+$

$F^{-}\cdots AsPh_4^+$			
As	0.43867774261091	0.24463289569463	0.13702413915519
F	0.49379562711073	0.09157461283060	-1.70793658098090
C	-0.53339705900547	-1.43619858165259	0.09300351276138
C	-0.22761324983728	-2.42079434219850	-0.84318315241929
C	-1.56603175670153	-1.65774264295840	0.99961211607244
C	-0.92262068339603	-3.62027154813054	-0.84452670508398
C	-2.28784289307182	-2.84236298797943	0.96569365905557
C	-1.95981617353740	-3.83075537442540	0.05174524208653
C	0.34351084126905	0.33198918399235	2.14946766053943
C	-0.33954984875030	1.31179678201860	2.86113167884850
C	0.96567509605356	-0.68971901666133	2.87022410132965
C	-0.38934424175215	1.28294634986286	4.25228528511861
C	0.92010694669954	-0.72379450211233	4.25342566853529
C	0.24133890936253	0.26832311133815	4.95076885880537
C	-0.52609742622286	1.87574617205314	-0.18679619064425
C	0.07033341246612	3.10281743802448	0.07627874085201
C	-1.81221110758357	1.83016488174719	-0.71009658763599
C	-0.62251589905176	4.27873365304469	-0.17326543747177
C	-2.50684293436122	3.00764340766534	-0.94332344555699
C	-1.91298360142935	4.23289367069265	-0.67829000777665
C	2.37771674691495	0.36581342333432	0.09363386648737
C	3.11038774172450	-0.16620008211471	-0.96582558593499
C	3.05467040482627	1.01940221867289	1.12104760795674
C	4.49360106401408	-0.07181174580381	-0.97487189290348
C	4.43629436472628	1.14128487147973	1.09019230120107
C	5.16004054310731	0.58583559154373	0.04777207030406
H	0.54978186939768	-2.26341509646065	-1.57489993578186
H	-1.81755988608462	-0.91334482739374	1.74354234264397
H	-0.66099516388619	-4.38672453668311	-1.56219683181193
H	-3.10000608566279	-2.99345049094485	1.66440714412287
H	-2.51052801979127	-4.76219123549992	0.03684720889447
H	-0.84891615312134	2.11393792730570	2.34284125490818
H	1.49918454764070	-1.47691901548863	2.34769512777796
H	-0.92579893097031	2.05762565016113	4.78536343715902
H	1.41250002703481	-1.52468580898447	4.78999491235908
H	0.20370122851259	0.24504084503389	6.03217073314715
H	1.07689934240438	3.15567456220163	0.47415549551744
H	-2.27987345309406	0.88109055125451	-0.94107058293251
H	-0.15031889168798	5.23166487557063	0.02676439571706
H	-3.51078013407283	2.96692052243338	-1.34495383151599
H	-2.45369174735883	5.15053146353758	-0.86907639820846
H	2.60878249873422	-0.64485144181278	-1.79178045468029
H	2.51612021554281	1.43816213898416	1.96003650920003
H	5.05087229999108	-0.50327858217429	-1.79598454428336
H	4.94383599920621	1.66397354804417	1.89014282244018
H	6.23901787108059	0.66764151095727	0.03016027262599

Table S11. XYZ coordinates of the optimized geometries of $SbPh_4^+$

	**$SbPh_4^+$**		
Sb	0.89839901709850	-0.38890089720279	-0.00857202079164
C	1.74701209495753	0.52540396545313	1.67150386069375
C	1.84628000921574	-0.19426319793559	2.85992149901183
C	2.18895883323555	1.84203630529708	1.59437585207974
C	2.39186205955796	0.41676111556345	3.97860506880831
C	2.73241509158859	2.44147452619636	2.72031964851442
C	2.83196734038126	1.73086557670327	3.90792177922892
C	1.54871012284523	-2.37673578560430	-0.04630459902953
C	0.67517852171788	-3.37783988408179	-0.45940686569441
C	2.85299758974677	-2.67879973974291	0.33676043724064
C	1.11822271384804	-4.69104369307980	-0.49539704194778
C	3.28339391363707	-3.99639693726258	0.29735917708297
C	2.41819907421949	-4.99780169271020	-0.12021750448304
C	-1.19042779176829	-0.32161157519585	0.09812496328197
C	-1.93707336336006	-0.51729057146961	-1.06102626807457
C	-1.81462657631695	-0.10801112119538	1.32276804728740
C	-3.32153037603222	-0.50457004489680	-0.98593823139253
C	-3.19994521370260	-0.09450742061023	1.38375915301787
C	-3.94961201959957	-0.29529674602634	0.23368121094376
C	1.48097823682132	0.61446298068966	-1.74981012037524
C	0.79588295691453	1.76721103889028	-2.12560206902067
C	2.55825141024788	0.14643354387956	-2.49533922879929
C	1.20068194460047	2.45719253945359	-3.25817197602221
C	2.95243217875015	0.84529899972786	-3.62638482829293
C	2.27759284605164	1.99788611773719	-4.00331919100811
H	1.51643774487627	-1.22456312263954	2.92496538908911
H	2.12748707944854	2.40196419905840	0.66901868483026
H	2.47464310640069	-0.13551442261071	4.90479288978504
H	3.08165780555541	3.46351764042772	2.66769455097688
H	3.25876960123096	2.20244270649312	4.78275708684643
H	-0.34668141462492	-3.15236420541585	-0.73985803922975
H	3.53388471580913	-1.90735467668990	0.67664367621526
H	0.44464518885098	-5.47548504556650	-0.81210209345489
H	4.29360816110228	-4.24014336139479	0.59665402670075
H	2.75733205609161	-6.02446707495766	-0.14740642368400
H	-1.45814973629949	-0.66700965214150	-2.02168415266888
H	-1.23930568896179	0.05899687774236	2.22546596735749
H	-3.90878464922506	-0.65434796672298	-1.88160328303065
H	-3.69314317666942	0.07571257632092	2.33099670090489
H	-5.02976110475543	-0.28325751423582	0.28721359848222
H	-0.05337241547489	2.12896308302772	-1.55783158912634
H	3.08564936855637	-0.75745852054602	-2.21500892724147
H	0.67348345062887	3.35232575066146	-3.55852974926444
H	3.78676900904009	0.48708050715013	-4.21367046758936
H	2.59014028376388	2.53853481946219	-4.88630859815818

Table S12. XYZ coordinates of the optimized geometries of $F^{-}\cdots SbPh_4^{+}$

	**$F^{-}\cdots SbPh_4^{+}$**		
Sb	0.41054449861346	0.20536715013113	0.11673831521516
F	0.44800276860880	-0.03073159898261	-1.86873452369355
C	-0.63988045332311	-1.65466000182094	0.12195592082128
C	-0.56327183649278	-2.51666307625672	-0.96936053016699
C	-1.42271217141639	-2.00216282610437	1.21853628291204
C	-1.24716292960873	-3.72336465439541	-0.94695600344095
C	-2.12320000928839	-3.20060663128280	1.22436254259011
C	-2.03067958313319	-4.06525538057710	0.14539157374986
C	0.31854979835542	0.36805627414026	2.30415436998604
C	-0.52740904553567	1.24797553011517	2.97236283649888
C	1.06834844658167	-0.53042466261386	3.06417044036220
C	-0.60913002861257	1.24521388286928	4.36090246344574
C	0.99003985849978	-0.53897895866044	4.44836922221994
C	0.15107889775151	0.35439423510533	5.10104794490318
C	-0.57951549798822	2.03562804554140	-0.27799391358391
C	-0.12544594935530	3.20658484331107	0.31823704994325
C	-1.66252476242724	2.07786051589040	-1.14797463538025
C	-0.76294771728381	4.41193108901941	0.05985848833860
C	-2.30849339036058	3.28165233339763	-1.38959854263282
C	-1.86066428225665	4.44801945223548	-0.78657284113459
C	2.53359231269908	0.32755379753364	0.03700225580138
C	3.22966159506177	-0.13447684985121	-1.07621509449034
C	3.22927285167231	0.93053847279262	1.08058957027497
C	4.61119970612134	-0.01865544559518	-1.12522911296584
C	4.60953086007688	1.06220540472849	1.01682644550807
C	5.30218436597292	0.58142115197939	-0.08301075382678
H	0.01785534510406	-2.24800931084589	-1.83783219324322
H	-1.49546492684632	-1.34934817818898	2.07782755930237
H	-1.17194911948620	-4.39470574720686	-1.79248126259964
H	-2.73971321777952	-3.45592937305314	2.07624868478489
H	-2.56997450253208	-5.00353706683876	0.15411939635248
H	-1.14177527724703	1.94708297203071	2.41803235960864
H	1.72753379871092	-1.24088304284240	2.57642059210079
H	-1.27172150978665	1.93993827823998	4.86140779259538
H	1.58316819326744	-1.24295466859722	5.01803205191720
H	0.08769563148905	0.35076347017127	6.18148730652597
H	0.72814083725092	3.19433832348753	0.98641392208616
H	-2.00398807401866	1.18033714577475	-1.64626739295097
H	-0.40065219588830	5.32140754507058	0.52109827355497
H	-3.15762624257867	3.30967661255627	-2.05983045128324
H	-2.36267593583206	5.38654254441874	-0.98190624326114
H	2.69869429563379	-0.57085272239082	-1.90885057830148
H	2.70721398529313	1.29675518028666	1.95486314186039
H	5.14801308768435	-0.39077382707662	-1.98809106051173
H	5.14059389112342	1.54003269178569	1.82973016145828
H	6.37917363350608	0.67704708056842	-0.12993183125079

Table S13. XYZ coordinates of the optimized geometries of $BiPh_4^+$

$BiPh_4^+$			
Bi	0.89997210426634	-0.38066129856530	-0.02035573971165
C	1.79427239706824	0.56324743453209	1.73965889974918
C	1.88486396698463	-0.16813513518043	2.91721959352585
C	2.25329108641381	1.86998777540719	1.65592448910928
C	2.44815834207319	0.43118678407329	4.03501657745323
C	2.81381908757404	2.45522109268979	2.78232033615293
C	2.90915872814646	1.73800866031914	3.96639252580345
C	1.57936501530633	-2.46024586258308	-0.04538703793477
C	0.70615195623928	-3.45047229641758	-0.47409132908183
C	2.86719289095386	-2.75795676018447	0.38021289461111
C	1.14108982855844	-4.76789389421071	-0.48210140092554
C	3.28709467039905	-4.08087978059749	0.36773369991332
C	2.42683170085468	-5.08003835909793	-0.06425097161093
C	-1.28577214164033	-0.32441216643547	0.08828694945611
C	-2.02056125027244	-0.51845339793831	-1.07407798457320
C	-1.90219230188271	-0.11800614115032	1.31436315857017
C	-3.40642569722599	-0.50970656436007	-0.99910835448443
C	-3.28870314280131	-0.10926481401766	1.37246995024872
C	-4.03626108572404	-0.30737596209464	0.22043100184309
C	1.50244379422989	0.68117176369513	-1.83682370698529
C	0.79090013536177	1.81414550251840	-2.20897757653567
C	2.59098167661499	0.22817332150885	-2.56891140476042
C	1.18654842245596	2.50942485545151	-3.34316858661693
C	2.97370204241846	0.93414670097251	-3.70079909628711
C	2.27563872243117	2.07125163907858	-4.08285927034461
H	1.53754224325997	-1.19269229240065	2.97820353505794
H	2.19182238211505	2.43322146411116	0.73274575847617
H	2.52694360399956	-0.12599530497958	4.95872710671033
H	3.17851918735393	3.47205124507746	2.73172391185308
H	3.34830905159193	2.19931240378813	4.84051168947423
H	-0.30441275917788	-3.21858211921630	-0.78788372324008
H	3.54214418438965	-1.98634991817761	0.73054366000566
H	0.47124029107143	-5.55064552342786	-0.81101859363802
H	4.28626333003356	-4.32874194011893	0.69932277675333
H	2.75844471633811	-6.10943656682774	-0.07008621420950
H	-1.53817992551628	-0.66417295487470	-2.03331627512209
H	-1.32685048603878	0.04523320681159	2.21759459067287
H	-3.99203729257758	-0.65838036133270	-1.89614534985774
H	-3.78356142405987	0.05451192922986	2.32009258204771
H	-5.11643371793257	-0.29954359231982	0.27289962661984
H	-0.06646125311832	2.15791474414713	-1.64276501529637
H	3.13596796835401	-0.66297089797046	-2.28219202492005
H	0.64193918907620	3.39294332863913	-3.64712547226140
H	3.81827473811068	0.59271846482278	-4.28362330726331
H	2.58047502392338	2.61667158760605	-4.96551687844670

Table S14. XYZ coordinates of the optimized geometries of $F^{-}\cdots BiPh_4^{+}$

$F^{-}\cdots BiPh_4^{+}$			
Bi	0.37945742373251	0.21553390232138	0.13938957606120
F	0.40963540193419	-0.03765387739266	-1.97702680921932
C	-0.70116595231773	-1.73217120565196	0.12522385073282
C	-0.96309363236097	-2.37619173587693	-1.07670941371757
C	-1.09780078075638	-2.28890720242930	1.33207213947180
C	-1.62824566095350	-3.59460176316909	-1.05916633412169
C	-1.76212592784102	-3.50877148183005	1.33623914595970
C	-2.02722930172844	-4.16144648885752	0.14254872005012
C	0.28720167143895	0.40903542201735	2.43335496813386
C	-0.80889650470108	1.00404029606909	3.05105585851505
C	1.25433371554785	-0.19659800686101	3.23071270089607
C	-0.92299234981410	1.01977272393486	4.43588602541047
C	1.14427793739488	-0.18394231389929	4.61562758307663
C	0.05696530427037	0.42950805540544	5.22016210106401
C	-0.60290192610198	2.15078881904359	-0.29443749255811
C	-0.43029249747755	3.20499903913812	0.59103202119882
C	-1.33309653681566	2.31698719551146	-1.46221275794299
C	-1.00928863130875	4.43641594899392	0.31328852157774
C	-1.92241430336267	3.54642288705635	-1.72274626431388
C	-1.76104106787908	4.60423061094130	-0.83936730288648
C	2.58570536654584	0.28578196050332	-0.00455915872955
C	3.23297296644029	-0.36971778581939	-1.04220981237686
C	3.30292172259511	1.02505938759812	0.92500426849222
C	4.61730353353620	-0.30946892317104	-1.12261174899150
C	4.68674230938409	1.09153065057449	0.82824150699067
C	5.34328434571379	0.41867172249679	-0.19097042322534
H	-0.65047579483967	-1.93187528152272	-2.00920179088593
H	-0.89847385833383	-1.79012478533207	2.27101461017358
H	-1.83515399106511	-4.10102502820105	-1.99307852704745
H	-2.07166574664780	-3.94498637314831	2.27699167404401
H	-2.54595938310143	-5.11119879320322	0.14810661281159
H	-1.59270944452366	1.46259945683617	2.45812965659083
H	2.10751169470150	-0.69174755267660	2.78056238654378
H	-1.77996532280229	1.49064578065052	4.90072713648546
H	1.90739529530560	-0.65626715961716	5.22116616999882
H	-0.02985212040281	0.44197276171957	6.29887293885851
H	0.14961912982500	3.08275092093567	1.49771198065444
H	-1.42730385251189	1.50647241942365	-2.16998669265498
H	-0.87027700878816	5.26176024941819	0.99938086529848
H	-2.50065593380301	3.68023262347372	-2.62770212598373
H	-2.21702879606867	5.56214262651122	-1.05270020501524
H	2.66666679169777	-0.90686379206575	-1.78921061524755
H	2.80086559647418	1.54996467496590	1.72858789046383
H	5.12869169943478	-0.82507950958832	-1.92495128934260
H	5.24810138962534	1.67055888185217	1.54991426300389
H	6.42196303070892	0.46611004292105	-0.26280640829771

Table S15. XYZ coordinates of the optimized geometry of PPh_3Cat

	**PPh_3Cat**		
P	1.23961295742041	-0.09673264491064	-0.02145459101928
Cl	-4.92934223675833	2.06817222190244	0.08360386585597
Cl	-2.02058793139983	3.27778198352369	-0.01013191587537
Cl	-2.77948835166004	-2.90563704238768	0.01951319512462
Cl	-5.29909629770615	-1.02529519085925	0.09170522586872
O	-0.20459075256792	-1.27161810632155	-0.08563539977850
O	0.08177995059606	1.10902252354390	-0.09042733304427
C	2.58056775992750	1.20770915630541	-0.05154663235556
C	-1.21145531688217	0.73124334933534	-0.04548954091722
C	1.69147447835835	-0.82495820034980	1.59745216040078
C	-1.35371418042630	-0.64807152949008	-0.04038360845072
C	-3.72785487012297	-0.34983553733358	0.04132905599256
C	-2.61893918503887	-1.20285703139969	0.00667598151359
C	2.38054274838494	2.58326595198564	0.02758300354099
C	-3.56705555277335	1.03230151168019	0.03650580940115
C	-2.28146362242230	1.58687159511385	-0.00564657473508
C	3.89219373250897	0.73429566652957	-0.13147323763499
C	1.79964237903117	-0.91962252575808	-1.55061649451793
C	1.99692573426477	-2.29448139193813	-1.63710888438462
C	1.43034256262910	-2.16094569720847	1.91580355780978
C	2.27851837042613	-0.01185488671164	2.56731400265057
C	1.99239550211107	-0.12868758630204	-2.67924267143312
C	3.46300472978674	3.45657735034195	0.02579003023771
C	1.79426080534348	-2.67332184778689	3.14966144413912
C	2.37715953340953	-1.85466748906909	4.10507364587095
C	4.75684884010790	2.97159671206654	-0.04971585668450
C	4.96971018079998	1.60145127720363	-0.12732821300097
C	2.60340483805760	-0.51936342775097	3.81555123615738
C	2.36742719294768	-0.70703813742820	-3.88191321037122
C	2.40726774091558	-2.86354955109464	-2.83158166034062
C	2.58467997192897	-2.07374840116569	-3.95795972889208
H	1.38388307123191	2.99435035904385	0.09387684043812
H	4.08225140767730	-0.33117300785620	-0.19372332551645
H	1.84880205165018	-2.93125575870888	-0.77683670338761
H	0.92756398701988	-2.80942802240956	1.21560870199146
H	2.48499074875464	1.02843159979827	2.36072170370416
H	1.85860990895367	0.94437703840153	-2.62563841113057
H	3.28597498335817	4.52254206100819	0.08530274012107
H	1.60208366969235	-3.71486526393190	3.36965118754878
H	2.64618477992251	-2.25499778741343	5.07368423313177
H	5.59675319152920	3.65393499573984	-0.04912942608926
H	5.97610856192551	1.20849792439402	-0.18713632694391
H	3.04204051805448	0.13361779656745	4.55785017196499
H	2.50032576250147	-0.08445849442802	-4.75640089037935
H	2.57763027305870	-3.93057479132211	-2.88393683033258
H	2.89276237347231	-2.52354772314913	-4.89249332624848

Table S16. XYZ coordinates of the optimized geometry of $F^{-}\cdots PPh_3Cat$ (F *trans* to Ph)

$F^{-}\cdots PPh_3Cat$ (F *trans* to Ph)			
P	0.03271432736720	-0.06835557225751	0.02684067747881
Cl	-0.00008014372395	0.25802915230792	4.83483517411384
Cl	2.91009883584277	-0.09338547668006	5.97056760516574
Cl	5.27679117375000	-0.64420709731532	4.00476965227874
Cl	4.71215812936964	-0.85052354921895	0.91558961456216
F	0.51093744766415	1.54169755634961	0.13808658318099
O	-0.07450873307152	-0.00513186086901	1.84035564736728
O	1.80373080696798	-0.45721876694759	0.28331541881077
C	-2.15576052025174	1.74272339588562	0.36736367764194
C	-1.78111497097299	0.45531520258622	-0.02518584145515
C	-2.80469489112254	-0.39130446500131	-0.45141331072559
C	-4.12706058156376	0.02768119211365	-0.51640775370932
C	-4.47216211009963	1.31224081682306	-0.13365419135867
C	-3.47578443023538	2.16444167183029	0.31674835003312
C	0.36092191511469	0.02672147623039	-1.82878179071078
C	1.49962324848288	0.66856076262561	-2.31855056457955
C	1.73958832112577	0.77627576644409	-3.68071579374092
C	0.84075160794897	0.25497159998799	-4.59902801871913
C	-0.29567820263089	-0.38500428620344	-4.13309546258731
C	-0.52439012740554	-0.49497791685119	-2.76840539145035
C	2.15476744968250	-0.38875277121236	1.53983841805145
C	3.43639589243278	-0.54394139618780	2.02716259596377
C	3.67448435495518	-0.45285321142891	3.40608551498550
C	2.62701631129234	-0.20623164336768	4.27631346099496
C	1.32453243895085	-0.04566125575357	3.78127470834249
C	1.09007474277924	-0.13407015275864	2.42489365518035
C	-0.63211048540503	-4.72909517717311	0.24873638218058
C	0.20903238333034	-4.18087116529920	-0.70706845334169
C	0.38749635719275	-2.80614772594299	-0.77753429624872
C	-0.27504080474553	-1.93941565497031	0.09042915594626
C	-1.09740165984123	-2.51224795510643	1.06156390434312
C	-1.28224101888941	-3.88572382256252	1.13618768690144
H	-1.40808643053327	2.43327940558082	0.72393655063230
H	-2.58630676340360	-1.41163405523793	-0.73450661023519
H	-4.88744103122012	-0.66225755515882	-0.86148131202237
H	-5.50240571619600	1.64337934562522	-0.17962562534858
H	-3.72400284315626	3.17066984565329	0.63216953246408
H	2.22457983701378	1.08086657961478	-1.63246857293643
H	2.63668946872308	1.27680682729112	-4.02461961027795
H	1.02554114183923	0.34389509388846	-5.66261110825214
H	-1.01159424873049	-0.80372492185452	-4.82978387526332
H	-1.41655726132740	-1.01054780319585	-2.44480962292601
H	-0.77240114818898	-5.80151968510086	0.30731589611622
H	0.73972441026022	-4.82406222447387	-1.39850247238537
H	1.06175039774955	-2.41031717099971	-1.52509234112307
H	-1.61334869486972	-1.87956442073017	1.77092077574250
H	-1.93238818225092	-4.29551693097844	1.89956138091924

Table S17. XYZ coordinates of the optimized geometry of $F^{-}\cdots PPh_3Cat$ (F *trans* to Cat)

$F^{-}\cdots PPh_3Cat$ (F *trans* to Cat)			
P	1.71886296270863	-0.65523073448240	-0.03741171281204
Cl	-4.50454281482155	1.45390580246339	0.25632599374963
Cl	-1.62916449902515	2.71843583091248	-0.00064950036465
Cl	-2.24852093718944	-3.47304187041475	0.20574025961180
Cl	-4.80701904635617	-1.65511342575065	0.34628244987042
F	2.71112854620274	-1.98241582307783	0.04128562295671
O	0.27282068168260	-1.84480510612659	-0.03743524764144
O	0.50396723742544	0.63874537385133	-0.13868660545924
C	3.12691970660599	0.59851410307666	-0.01719991798500
C	-0.73521604383043	0.20256773938071	-0.02734403324520
C	1.57345664304261	-0.68212094718506	1.85622144043305
C	-0.85581965585933	-1.19810904759265	0.02461030705909
C	-3.24825867752100	-0.93940265646908	0.20786389047983
C	-2.11498433277072	-1.76107754624606	0.14186274367073
C	2.92575010677511	1.98137065711291	-0.01220148533005
C	-3.11845901726640	0.43845894934635	0.16395301945381
C	-1.84497431360008	1.01246836918381	0.04866707494444
C	4.44824509606807	0.14866908732445	0.00219235517141
C	1.72031262058901	-0.81619355587757	-1.92443803492991
C	1.74718257826583	-2.06566996900732	-2.54504277775086
C	1.69997218994260	-1.87347784090730	2.57241780367611
C	1.32870187521862	0.47721931676713	2.58947003792383
C	1.70428334968352	0.30653480692088	-2.74873254307651
C	3.99243759562833	2.86919766512757	0.01593365655172
C	1.60205332596674	-1.90089603210432	3.95544348808280
C	1.36769696140368	-0.73461213997838	4.66798588537582
C	5.29645649579910	2.40193103932513	0.03848835477158
C	5.51618910871554	1.03366342776238	0.02984608391705
C	1.22819177375088	0.45579527360977	3.97433727116557
C	1.72123567234784	0.19315000740102	-4.13239786791138
C	1.76171725506742	-2.18574618413490	-3.92687561020852
C	1.75088893291450	-1.05588928406172	-4.73111561378126
H	1.92354420353627	2.38580613010159	-0.03496033216687
H	4.65833352029317	-0.91034567702552	-0.00515938592915
H	1.74113920168871	-2.96607701868497	-1.94796258042016
H	1.86303496895686	-2.80415240308527	2.04939440551221
H	1.21327825679171	1.42636516100450	2.08522088629459
H	1.68464561109778	1.29700905333967	-2.31557601838937
H	3.79691958254577	3.93481542003710	0.01976472326831
H	1.70403048716950	-2.84442108136754	4.47832838455447
H	1.28830738049460	-0.75579269504465	5.74800054355922
H	6.12956467995583	3.09367734694988	0.06122657626866
H	6.52792139621894	0.64639005745357	0.04498990874363
H	1.03504706928955	1.37808877255313	4.50866333529985
H	1.70860681675522	1.08902850468766	-4.74130149045274
H	1.77807168774925	-3.17117538754366	-4.37694719663970
H	1.76151375989221	-1.14876146952485	-5.81026854787231

Table S18. XYZ coordinates of the optimized geometry of $AsPh_3Cat$

	$AsPh_3Cat$		
As	1.19819314927206	-0.07524048677052	0.01697935961537
Cl	-5.08077610540807	2.09981097991558	-0.35177037837814
Cl	-2.17594223299611	3.31012234927221	-0.37509778704136
Cl	-2.92417934610297	-2.85144982044215	0.06890063280618
Cl	-5.44995705647784	-0.98880523642882	-0.12629321826055
O	-0.36697126976883	-1.26634078690286	-0.04218032638643
O	-0.07345693161469	1.19748705872365	-0.20583146809770
C	2.55118696861960	1.34852862568817	-0.06787410744379
C	-1.34740183539265	0.77776374581140	-0.19471549996641
C	1.61335390019304	-0.77169096486499	1.77506935803866
C	-1.49473449990737	-0.60435434906641	-0.09971103076571
C	-3.87871523056656	-0.30940001482753	-0.15105462767919
C	-2.76572796073972	-1.15157142869831	-0.06975426445235
C	2.27816270230249	2.70419595311819	0.09591041945994
C	-3.71752413287772	1.06794855941917	-0.24982986706599
C	-2.43152416740171	1.61974522558957	-0.26601236220776
C	3.87599654224071	0.94422110704094	-0.23569683393875
C	1.84032663258543	-1.09440447295128	-1.48374660570775
C	1.95663054634943	-2.47890822589825	-1.42064867420987
C	1.10447489022559	-1.99471801380298	2.21790719136406
C	2.44435756601348	-0.03580981204357	2.61763280838017
C	2.15078777207396	-0.42152892141217	-2.65985105256394
C	3.31207247051959	3.63217275422716	0.08702287057138
C	1.45099242904920	-2.47761581542393	3.46959812406836
C	2.28178853568630	-1.74277842501340	4.30164872872455
C	4.62448123677988	3.22062555566422	-0.07832982586902
C	4.90552275747418	1.87136436624589	-0.23912512344853
C	2.76952244852324	-0.51812023462323	3.87642413824905
C	2.56993455868263	-1.13485801280130	-3.77323597509086
C	2.40216290626611	-3.18220287540200	-2.52825349960684
C	2.70134389759340	-2.51298781985201	-3.70637840238036
H	1.26485319380885	3.05451231943583	0.22759262559995
H	4.12133836940917	-0.10424368011177	-0.36182115418566
H	1.71217394538779	-3.01880606861636	-0.51579834515743
H	0.42526187227199	-2.56778344392684	1.60547831510476
H	2.84260953423698	0.92082715417108	2.31133045505948
H	2.07316245803906	0.65715882441825	-2.71844364640797
H	3.08464902731703	4.68285389721839	0.21062178045430
H	1.05679075615446	-3.43001863323615	3.79790247014444
H	2.54128952582521	-2.12097745954406	5.28168507751555
H	5.42637696164362	3.94720048850191	-0.08338912871299
H	5.92695433780176	1.53889688929019	-0.36918572864035
H	3.40755130329674	0.06942206189010	4.52274568682270
H	2.79861549343817	-0.60953183369204	-4.69075005580022
H	2.50570770093967	-4.25755389826434	-2.47285666630261
H	3.03748737923334	-3.06718818102465	-4.57267138621039

Table S19. XYZ coordinates of the optimized geometry of $F^{-}\cdots AsPh_3Cat$ (F *trans* to Ph)

$F^{-}\cdots AsPh_3Cat$ (F *trans* to Ph)			
As	0.01109967825219	-0.08382425195273	0.02037325384814
Cl	0.08711923498868	0.29806200173442	4.91009419189757
Cl	3.01828841180338	0.07571433606265	6.01195202629172
Cl	5.37997216501748	-0.42219489659255	4.02348514530725
Cl	4.77820212750843	-0.72238995803659	0.95162638966659
F	0.51896945668214	1.63757064185116	0.17442586021149
O	-0.04769717657318	-0.04026720052410	1.95368705772299
O	1.88390497510540	-0.47799759748428	0.33385171435386
C	-2.25361637408101	1.73809522688199	0.45888400075573
C	-1.88717002007573	0.47230839245227	0.00337542814174
C	-2.89549671370169	-0.36483008557352	-0.46837406968653
C	-4.21991638572166	0.05198969938575	-0.51125440174022
C	-4.56654142656026	1.31568188481323	-0.06336706794626
C	-3.57646677359915	2.15336951969068	0.42867180695251
C	0.40657299651792	0.03337208146437	-1.91335153298990
C	1.59023315685826	0.61577299634206	-2.36190331944041
C	1.86741159726112	0.70952263128294	-3.71835560563708
C	0.96247241733912	0.23513310101042	-4.65663618611143
C	-0.22182849051535	-0.33909095593006	-4.22391444264942
C	-0.49174215518780	-0.43742204404966	-2.86534962553068
C	2.21116467549748	-0.35592725155431	1.59264923672690
C	3.50392248050892	-0.44285513855926	2.07476954888764
C	3.76208334710996	-0.30959368084872	3.44518004137884
C	2.71758593591839	-0.08420046691906	4.32389489409579
C	1.40591613321566	0.01567096059633	3.84064081507641
C	1.14354657436838	-0.11770037927869	2.48966816985047
C	-0.72553501643782	-4.82195176117491	0.18264312934110
C	0.12178605282827	-4.27538292386140	-0.76895087336968
C	0.32559868033478	-2.90245997544327	-0.81705049330196
C	-0.31826042368276	-2.04893836338949	0.07398442959255
C	-1.14920516040058	-2.61342462755923	1.03974128131124
C	-1.35781656297865	-3.98474430811230	1.08981604393200
H	-1.50089580451930	2.40882912864741	0.84413353823731
H	-2.66452956807860	-1.36868232908090	-0.80129401272862
H	-4.98019263144863	-0.62019678315596	-0.88965817409541
H	-5.59841295771858	1.64346115140338	-0.09177547481422
H	-3.83322848369678	3.14103764593885	0.79177176445813
H	2.31526562215729	0.98601788567560	-1.65154392600795
H	2.79779946473949	1.15940409898396	-4.04279611826028
H	1.17952733163387	0.31111378656900	-5.71506601278093
H	-0.94039114478167	-0.71481899298703	-4.94192595910394
H	-1.42192750641825	-0.89774021693895	-2.56043117405745
H	-0.88611212655281	-5.89234703408865	0.22244189591281
H	0.63438688746747	-4.91811636684599	-1.47424767585112
H	0.99988234202087	-2.50189063216041	-1.56328748381479
H	-1.64672457705708	-1.98081552794866	1.76324441789451
H	-2.01216426534763	-4.39903342073574	1.84704754807299

Table S20. XYZ coordinates of the optimized geometry of $F^{-}\cdots AsPh_3Cat$ (F *trans* to Cat)

$F^{-}\cdots AsPh_3Cat$ (F *trans* to Cat)			
As	1.69333428338725	-0.67192359598798	0.06085087629364
Cl	-4.63551227639672	1.50817824203672	-0.12256250948151
Cl	-1.74132340371290	2.73198462503466	-0.28905515256213
Cl	-2.43089435754898	-3.41866832719235	0.32182180475207
Cl	-4.97372958843241	-1.58462064970182	0.18367149864860
F	2.74826651969765	-2.10127535314179	0.25327757133190
O	0.10090072995590	-1.87916202126387	0.10406503822103
O	0.37611854448481	0.68501196599392	-0.16201913123549
C	3.15220115965078	0.67356850315436	0.02894177452037
C	-0.85594895449984	0.22135095718203	-0.08297379971306
C	1.49323575400142	-0.60630619886541	2.03501409028972
C	-0.99509728144903	-1.18023622363454	0.05970788673020
C	-3.40212342180392	-0.88868856405363	0.08700783676958
C	-2.27379209058824	-1.71462912925011	0.14563216431066
C	2.91387999201334	2.04528982274975	-0.06066734207356
C	-3.25608306484071	0.48067048561147	-0.04953737756416
C	-1.97128434430567	1.03246567534732	-0.12904794894289
C	4.47448799870569	0.24323166917722	0.12310681153646
C	1.71948183506326	-1.00123306732550	-1.89113369867974
C	1.68296209376859	-2.29437928216370	-2.40575558602147
C	1.58289370176956	-1.76117442088372	2.80780842763123
C	1.27134181877073	0.60408324689235	2.68334524573243
C	1.78996985032463	0.06344957869472	-2.78183249450871
C	3.96331725503889	2.95475887805190	-0.05226978002873
C	1.47185159485246	-1.70223436657969	4.18948376048912
C	1.26155617842440	-0.48848567093393	4.82686546529689
C	5.27344206411674	2.51320969394024	0.04472017195612
C	5.52325233373122	1.15215415344371	0.13076553710583
C	1.15726181421575	0.66474722208971	4.06622098116311
C	1.83366722661662	-0.15406585102394	-4.15295880134815
C	1.72582533483912	-2.51402168298802	-3.77487945314308
C	1.80379056908614	-1.44477769322365	-4.65564581113672
H	1.90221832682411	2.42038484872507	-0.14034305735494
H	4.69739523982882	-0.81244772329921	0.19286295815361
H	1.61142039496896	-3.14254158950100	-1.74004654818249
H	1.73468090588949	-2.72084472615771	2.33584921590467
H	1.18522697460803	1.52189001168417	2.11747307564465
H	1.81823935769330	1.08162013579175	-2.41678447646337
H	3.75109321392521	4.01485517716503	-0.12128646491380
H	1.54678352756308	-2.61356613349988	4.77043060765859
H	1.17419755126441	-0.44340932324161	5.90549230021921
H	6.09204248668321	3.22239238910186	0.05310139387717
H	6.54214492512002	0.79187812071968	0.20600266490045
H	0.98328429770994	1.61975424745971	4.54674792615297
H	1.88920847476907	0.69195193873434	-4.82700416992796
H	1.69534333110697	-3.52802723703443	-4.15465808453569
H	1.83718112310879	-1.61696275783416	-5.72440539747259

Table S21. XYZ coordinates of the optimized geometry of $SbPh_3Cat$

	$SbPh_3Cat$		
Sb	3.14739978051197	0.78774056381514	2.43126525410415
Cl	5.82388467227563	-0.34534837555334	6.46045289397355
Cl	8.74556759097912	-1.25950770020890	5.76356054058456
Cl	9.65945641432586	-1.33336843266723	2.77839096285255
Cl	7.63503421782936	-0.52032026381963	0.51822975931126
O	4.98696274608738	0.26078028808272	1.59458679913573
O	4.23642546073395	0.34030101565774	4.06685523441878
C	5.88683863452606	-0.09726440801567	2.48723373666835
C	5.48823207810911	-0.05618798513328	3.82879320100812
C	6.36379058881648	-0.41175187799269	4.83465489599868
C	7.66271046840343	-0.81429346094646	4.51298332249760
C	8.06672759466022	-0.85099438721534	3.18392780450612
C	7.17582202206437	-0.49160294952068	2.17014667160587
C	1.55411653398269	1.31572171484184	3.76284269378323
C	0.31602863498988	1.59071838043245	3.18225132086374
C	-0.76395823110843	1.96163142733646	3.96881074952910
C	-0.61344594014037	2.06330929119539	5.34437216168770
C	0.61472934065372	1.79165338746193	5.92674084472269
C	1.69900123856315	1.41775122510863	5.14265789406147
C	3.25596464065935	2.59388707181561	1.31593256306353
C	2.51725009606667	3.69832403622967	1.72834628919043
C	2.54610026274895	4.86949746957228	0.98415679439953
C	3.30478883272806	4.93866628704136	-0.17343407184258
C	4.04938801638218	3.84076881255297	-0.57997279749219
C	4.03604560748727	2.67053251968731	0.16329013215093
C	2.23229401783977	-0.82757525377770	1.41630829959345
C	2.72967535604891	-1.26663203938689	0.19219417863311
C	2.10794309567034	-2.31831364585645	-0.46298414623729
C	1.00811150108683	-2.94418922416042	0.10618368334670
C	0.52512848756956	-2.51848573558698	1.33359162557356
C	1.13248506565437	-1.45705199621130	1.98920108436537
H	0.17802306787315	1.51740467530541	2.10836308354396
H	-1.71983445886689	2.17129991046711	3.50702120475974
H	-1.45377870584830	2.35405349698510	5.96093074792045
H	0.73455997175073	1.86891665464160	6.99930744219874
H	2.64649669911549	1.20372483512628	5.61631853764875
H	1.92118657195063	3.66463004109405	2.63072947503297
H	1.97515492249725	5.72708834742908	1.31424945941399
H	3.32308282482376	5.85052823031848	-0.75565577372547
H	4.65110847247733	3.89549940595660	-1.47745676363764
H	4.64771159560619	1.83762139719859	-0.15164642975026
H	3.60479849753284	-0.81078632248970	-0.24967359352903
H	2.49136129593228	-2.65542707241200	-1.41678900516325
H	0.53142603185105	-3.76951632398410	-0.40599566749787
H	-0.32460749303259	-3.01223514782251	1.78579074716659
H	0.74268088013112	-1.13493088259257	2.94637315956046

Table S22. XYZ coordinates of the optimized geometry of $F^{-}\cdots SbPh_3Cat$ (F *trans* to Ph)

$F^{-}\cdots SbPh_3Cat$ (F *trans* to Ph)			
Sb	0.03410398414455	0.02639462520580	0.02678228289109
Cl	0.12611867805021	0.23024313531699	5.05478121565981
Cl	3.03801846876070	0.01772764204726	6.19840928498637
Cl	5.44437062228817	-0.32982774576138	4.23052214172331
Cl	4.90038005239891	-0.49201729512158	1.14163218578433
F	0.46355067452901	1.92784631083794	0.17013894232244
O	0.02315874378550	0.06420873570242	2.11457722339623
O	2.04909337839941	-0.28527746345275	0.46299018367114
C	-2.49907257826230	1.78663870122542	0.37986348806720
C	-2.06204656244420	0.52080549703556	-0.00148510547536
C	-3.00596554870786	-0.41520486721993	-0.41411088770851
C	-4.35709345721395	-0.09371866502155	-0.45604666610469
C	-4.78197305375774	1.16800837439707	-0.07044284517195
C	-3.84957022225518	2.10500492679319	0.35202786347386
C	0.50370257046234	0.10609569549854	-2.07319401851173
C	1.82077856210967	0.11757028814764	-2.52754588142309
C	2.09748322845308	0.15876849731842	-3.88768713492724
C	1.06526832956411	0.19211349205078	-4.81390314012882
C	-0.24828256512209	0.18685724633687	-4.37114925514200
C	-0.52431454745948	0.14367543272408	-3.01042924447981
C	2.31428984635729	-0.20892241780511	1.74243145727955
C	3.59943721193691	-0.29446287605787	2.25244699709411
C	3.83456783279565	-0.22275552318890	3.62963634232308
C	2.77026967435790	-0.06339645963655	4.49930453555954
C	1.46856387021036	0.03429387757888	3.99498427742455
C	1.22337808999281	-0.03029830123959	2.63246594286854
C	-0.72131329381885	-4.89164208757502	0.15673508272091
C	-0.10059872959243	-4.30892617215615	-0.93734199799259
C	0.11548121397458	-2.93656074893610	-0.96689330382145
C	-0.28305156413134	-2.12598808504902	0.09143740547423
C	-0.89839755340705	-2.72449905225943	1.18944739264971
C	-1.11855346165679	-4.09479682691792	1.22110884709495
H	-1.78053461567476	2.52780789090994	0.69968929707650
H	-2.69566807944113	-1.41271220968266	-0.70254327169007
H	-5.07650702009135	-0.83391634090580	-0.78420493764642
H	-5.83513078140602	1.41964691403193	-0.09573847574367
H	-4.17452533618677	3.09125007752045	0.66013092609856
H	2.63943915130419	0.09247676757584	-1.82100116753433
H	3.12723397852447	0.16597795460701	-4.22346834344541
H	1.28373849436507	0.22505702396750	-5.87416688538129
H	-1.06311926181417	0.21829066287600	-5.08413856328414
H	-1.55884067649622	0.14780254417308	-2.68776903205261
H	-0.89182942465778	-5.96094810969190	0.18205731476843
H	0.21931769463484	-4.92173714209455	-1.77138528633782
H	0.60103326170152	-2.50211812024622	-1.83252467091141
H	-1.21054775922195	-2.12044990992768	2.03181010270971
H	-1.59900352028185	-4.54108889393095	2.08327438179623

Table S23. XYZ coordinates of the optimized geometry of $F^{-}\cdots SbPh_3Cat$ (F *trans* to Cat)

$F^{-}\cdots SbPh_3Cat$ (F *trans* to Cat)			
Sb	2.72947071466870	0.87033245981100	1.73807551249655
Cl	5.62785945588521	-0.03424204053057	5.72626916755521
Cl	8.51217314932448	-0.99483627142648	4.94521520709310
Cl	9.29584568980770	-1.20239948991475	1.92453772216116
Cl	7.16307108272919	-0.51864742555081	-0.27368647104585
F	1.76891830460255	1.06471967199205	0.05169058767166
O	4.57138344544703	0.28752247330538	0.83688780285145
O	3.93145566977012	0.54916167043569	3.41173667795859
C	5.48222583934076	0.00901605174350	1.73249208673397
C	5.14212078537492	0.13671090731146	3.10664389654223
C	6.08420559361701	-0.17912809807953	4.07173669372006
C	7.37010555851609	-0.60172287662540	3.71822388276737
C	7.71246794667317	-0.70409560448872	2.38165808701286
C	6.76663493077016	-0.39884861955827	1.39775067254810
C	1.06978796412746	1.41104979096290	3.01510506913335
C	-0.15130369759732	1.72526989275384	2.42315676119263
C	-1.24397720083187	2.08443062936624	3.20058825921349
C	-1.13069303792353	2.13465742931141	4.58220639982493
C	0.08103390130745	1.82370446344813	5.18134672970253
C	1.17514495305564	1.46444225474120	4.40367211800928
C	3.45199453394796	2.89177738212515	1.49179602335480
C	3.04851818402565	3.88271222369345	2.37911205274524
C	3.50040422107665	5.18949553854365	2.24098653115385
C	4.35786386838967	5.52309890541973	1.20498110019868
C	4.76166635402275	4.54202497459888	0.31075881244588
C	4.31608735658442	3.23550590023475	0.45486955612058
C	2.22299319846144	-1.21962743087709	1.63038929364501
C	2.54397374629222	-1.97612553207045	0.50715645141450
C	2.17342876650434	-3.31060382603311	0.42794995380620
C	1.48157250634474	-3.90790353915642	1.47248137019607
C	1.16532189427939	-3.16272030406103	2.59738200350753
C	1.53293735362910	-1.82484539946219	2.67251858867195
H	-0.25518522421883	1.69247371969375	1.34595448889069
H	-2.18602127213373	2.32756618391954	2.72422522674532
H	-1.98264023978239	2.41625468684174	5.18867242806236
H	0.17865767798756	1.86138926147291	6.25943188315027
H	2.11469520385109	1.22664707206009	4.88660523194330
H	2.37250630945584	3.64891042969860	3.19188291012720
H	3.18049742514655	5.94572854586834	2.94740085923784
H	4.71260411736864	6.54069464415775	1.09584063702699
H	5.43377675003932	4.79142092072248	-0.50124747042279
H	4.65795866370687	2.48236678554875	-0.24105553286578
H	3.09398680188570	-1.53091927661328	-0.31053932059009
H	2.42805483499690	-3.88626696559902	-0.45363714071819
H	1.19265137330201	-4.94979915934327	1.40917865745986
H	0.62935819427522	-3.62020454341317	3.41986101862805
H	1.26775635189586	-1.25603846697890	3.55542752292186

Table S24. XYZ coordinates of the optimized geometry of $BiPh_3Cat$

$BiPh_3Cat$			
Bi	2.98079297828678	0.54993407926785	2.39139397541857
Cl	5.98469736690295	0.29439875471782	6.39593635654408
Cl	9.01085998418956	-0.06577933008200	5.64469756826486
Cl	9.81170848454170	-0.43792874287322	2.64901262238728
Cl	7.57496242405065	-0.49721622339966	0.44814978394936
O	4.86193459300137	-0.27911080307305	1.56475113890981
O	4.19266287795112	0.09531764409628	4.08896299255971
C	5.83117848866533	-0.24551541479205	2.44865462133108
C	5.47972158534482	-0.05321524582794	3.79957819254644
C	6.46324555325381	0.02587174802402	4.76937643629325
C	7.80882848890761	-0.11462776653595	4.42458689368384
C	8.16306842894587	-0.28820068088650	3.09297277387007
C	7.17460760562500	-0.33222922128772	2.10979715664196
C	1.39229251604181	1.17153118167378	3.84276210666866
C	0.13895420075678	1.46800935670738	3.31739201720562
C	-0.87345469787641	1.91341891618342	4.15691942033304
C	-0.63073017719609	2.05920750774337	5.51465095516708
C	0.62138644649718	1.76276657965753	6.03271568253075
C	1.64177421859890	1.31948416655244	5.20057943040476
C	3.39324212056998	2.45442382457967	1.33699893036996
C	2.63104327653639	3.56503232522048	1.66962675311834
C	2.86527301156850	4.76598330888206	1.01388369449040
C	3.85332375629216	4.84621185851853	0.04408818072361
C	4.61502689258080	3.73009624166337	-0.27040714811513
C	4.39294179862189	2.52229748193254	0.37701510309126
C	1.94716512834822	-1.04327717822553	1.26434932083902
C	2.45655538412224	-1.47087527972419	0.04576310669351
C	1.77971427116783	-2.46098873887995	-0.65135923904285
C	0.62359542611298	-3.02180739885649	-0.12690440369925
C	0.13480425458266	-2.59617976500193	1.09849364780170
C	0.79642849753810	-1.59824667967461	1.80226342392801
H	-0.06722662578313	1.35585109665352	2.25800913982873
H	-1.84850961975716	2.14480187247353	3.74850710312469
H	-1.41934421508151	2.40495045203114	6.16977470658004
H	0.81024992721805	1.87686022175369	7.09199904649144
H	2.61461206554398	1.09392009156881	5.61450898036788
H	1.86692355582486	3.51409117801562	2.43569795471838
H	2.27777277586979	5.63835822578757	1.26762685919182
H	4.03598319323365	5.78446500423242	-0.46276039674398
H	5.39396846826155	3.79716051127504	-1.01821284204407
H	5.00394378942595	1.66146214506730	0.14146106443395
H	3.37205261318200	-1.06074703713703	-0.35670757028078
H	2.16522270534664	-2.80036596627480	-1.60354093313947
H	0.10583029535180	-3.79842122070994	-0.67385766822594
H	-0.75982655423148	-3.04104474843665	1.51326529922543
H	0.41061144106436	-1.27386133260002	2.76096476156301

Table S25. XYZ coordinates of the optimized geometry of $F^{-}\cdots BiPh_3Cat$ (F *trans* to Ph)

$F^{-}\cdots BiPh_3Cat$ (F *trans* to Ph)			
Bi	-0.00173047086818	0.01575880825333	0.00773845731932
Cl	0.13355701561773	0.17145456085808	5.12652791479896
Cl	3.03863061742674	0.00802076907775	6.29107975787584
Cl	5.46627217096213	-0.27209176175011	4.33564696373397
Cl	4.94716840536104	-0.41841598037841	1.24581340681739
F	0.43169573720859	2.02976508900070	0.15005803025787
O	0.03698951787685	0.02729812918012	2.21042679934060
O	2.11849807728903	-0.28044323244154	0.53578200140250
C	-2.57899309580215	1.88214784484813	0.12769153893172
C	-2.18214006491571	0.55812498647054	-0.01067138618936
C	-3.13467910284064	-0.43742928652876	-0.17697614432959
C	-4.48596744010012	-0.11150823765022	-0.20862578007170
C	-4.88362230385970	1.20937673474496	-0.06909121014161
C	-3.92959008664178	2.20333667609918	0.10081261728211
C	0.56086287723430	0.12044627054179	-2.16024824956436
C	1.88688508203649	0.07115704775322	-2.57120277482076
C	2.19131119460367	0.14655026027959	-3.92432839382005
C	1.17929506410047	0.27099346805046	-4.86607836240856
C	-0.14362131691271	0.32098612313173	-4.45380187482483
C	-0.45238923870897	0.24495485140548	-3.10072762721177
C	2.34795716836438	-0.19677316936842	1.81655493530048
C	3.63045047849187	-0.25278636747951	2.34630555883746
C	3.85785994173018	-0.18891215683746	3.72460590487116
C	2.78576563043522	-0.05850259953547	4.58810507616188
C	1.48817943487128	0.01728994483809	4.07182983543363
C	1.24353386265669	-0.04272010911955	2.70637009512103
C	-0.71103570529987	-4.99875620366262	0.11730362823098
C	-0.33166940405551	-4.36552013671003	-1.05646228888774
C	-0.13303009599576	-2.98960508593570	-1.07117954617946
C	-0.31253742105666	-2.24130020930900	0.08434036530868
C	-0.69061463714996	-2.87855627643758	1.26077021832099
C	-0.88971344400291	-4.25351764347870	1.27437674302431
H	-1.83437366914019	2.65435893699437	0.26082147948307
H	-2.83517645216651	-1.47461735992247	-0.27406561408953
H	-5.22541632752163	-0.89229141611557	-0.33779954101732
H	-5.93598225896113	1.46434749942661	-0.08891461125316
H	-4.23809209921215	3.23512641385480	0.21640320562077
H	2.68149414307375	-0.02127121845080	-1.84260156008715
H	3.22590646623688	0.11057779302171	-4.24253152994960
H	1.42246278069232	0.33273202603843	-5.91948583782303
H	-0.93784047656813	0.42302032498988	-5.18311851775216
H	-1.49034970847714	0.29295140338639	-2.78913162409577
H	-0.86362885893092	-6.07085247103236	0.13160180260457
H	-0.18539896291972	-4.94063144752559	-1.96274681430124
H	0.16946994108806	-2.50926700646458	-1.99479993474280
H	-0.82837963300849	-2.30786326551720	2.17004234947573
H	-1.18143533224106	-4.74384632059362	2.19509553800656

Table S26. XYZ coordinates of the optimized geometry of $F^{-}\cdots BiPh_3Cat$ (F *trans* to Cat)

$F^{-}\cdots BiPh_3Cat$ (F *trans* to Cat)			
Bi	2.59914181161580	0.71482019704681	1.67859608846044
Cl	5.70848382671248	0.16781987507118	5.67816771166585
Cl	8.74112217238946	0.00719696764383	4.89221729861312
Cl	9.54177221283296	-0.10546608533879	1.86896164091626
Cl	7.29599970557955	-0.08860880239431	-0.32132784907915
F	1.40116268012551	1.23666020989106	0.06664423910628
O	4.58938241830121	-0.03798438159697	0.78342535174645
O	3.90720818665172	0.13578254672561	3.38199217258783
C	5.53049295193452	-0.02167194763942	1.68188064341291
C	5.17092582786866	0.05974842588193	3.06371430788557
C	6.17794798217273	0.07408708028782	4.02072930357916
C	7.52958238434741	0.01080408567054	3.66771067240172
C	7.88252958235208	-0.04369993066336	2.33152573836705
C	6.88347316872851	-0.04582398657644	1.35375950789276
C	1.03310571964249	1.32540807557887	3.19211409649049
C	-0.17277277220417	1.83910396189517	2.72597444163312
C	-1.15201370940935	2.24655369086688	3.62372291447031
C	-0.92914453733953	2.14563513266103	4.98934386134990
C	0.27442654636814	1.63602353644463	5.45543099006154
C	1.25583949799814	1.22447463295464	4.56122692015551
C	3.58232352830003	2.71901769883158	1.47775504765338
C	3.13175829248821	3.73022872673996	2.31065438618549
C	3.70563448766055	4.99360678940047	2.23509321985941
C	4.72570594023445	5.24045370186068	1.32969510243595
C	5.17087429697223	4.22245490406427	0.49904226458435
C	4.60262152675941	2.95713062787930	0.56921076736926
C	1.90710153131762	-1.38418041657276	1.35552235806116
C	2.67043051776923	-2.33282036962309	0.69250225821411
C	2.16436450348065	-3.61550201609707	0.52203834360695
C	0.90927789074801	-3.94869774128375	1.00967914745482
C	0.15377623291045	-2.99389346068863	1.67278642501033
C	0.65130007062099	-1.70815248289458	1.84417556197516
H	-0.34698211386065	1.92667542708389	1.66044365083255
H	-2.08856304985084	2.64639804775891	3.25416432192208
H	-1.69114622813811	2.46585347349265	5.68913406040274
H	0.45377925999146	1.55909194974005	6.52082791592323
H	2.19440800814092	0.83422694838054	4.93371514610065
H	2.33863310167363	3.54899696393969	3.02546394622426
H	3.35578526318863	5.78106551454836	2.89102091641128
H	5.17815031194739	6.22274780208556	1.27514469269236
H	5.97385093985221	4.40589963071803	-0.20373661297403
H	4.96668567250364	2.16748019325546	-0.07277433420826
H	3.64977505822381	-2.07873458230536	0.31504319372236
H	2.76015160474752	-4.35736069811663	0.00502052200509
H	0.52246797959556	-4.95111050380960	0.87531577742034
H	-0.82581905924954	-3.24516421430317	2.06001491401302
H	0.05231877530395	-0.97310519849554	2.36746695538455
